# Amnionless-mediated glycosylation is crucial for cell surface targeting of cubilin in renal and intestinal cells

**DOI:** 10.1038/s41598-018-20731-4

**Published:** 2018-02-05

**Authors:** Tomohiro Udagawa, Yutaka Harita, Kenichiro Miura, Jun Mitsui, Koji L. Ode, Shinichi Morishita, Seiya Urae, Shoichiro Kanda, Yuko Kajiho, Haruko Tsurumi, Hiroki R. Ueda, Shoji Tsuji, Akihiko Saito, Akira Oka

**Affiliations:** 1 Department of Pediatrics, Graduate School of Medicine, The University of Tokyo, 7-3-1 Hongo, Bunkyo-ku, Tokyo, 113-8655 Japan; 2 Department of Pediatrics and Developmental Biology, Tokyo Medical and Dental University, 1-5-45 Yushima, Bunkyo-ku, Tokyo, 113-8519 Japan; 3 Department of Neurology, Graduate School of Medicine, The University of Tokyo, 7-3-1 Hongo, Bunkyo-ku, Tokyo, 113-8655 Japan; 4 Department of Systems Pharmacology, Graduate School of Medicine, The University of Tokyo, 7-3-1 Hongo, Bunkyo-ku, Tokyo, 113-8655 Japan; 5 Department of Computational Biology and Medical Sciences, Graduate School of Frontier Sciences, The University of Tokyo, 5-1-5, Kashiwanoha, Kashiwa-shi, Chiba, 277-8561 Japan; 6 Department of Applied Molecular Medicine, Niigata University Graduate School of Medical and Dental Sciences, 1-757, Asahimachidori, Chuo-ku, Niigata-shi, Niigata, 951-8510 Japan

## Abstract

Mutations in either cubilin (*CUBN*) or amnionless (*AMN*) genes cause Imerslund–Gräsbeck syndrome (IGS), a hereditary disease characterised by anaemia attributed to selective intestinal malabsorption of cobalamin and low-molecular weight proteinuria. Although cubilin protein does not have a transmembrane segment, it functions as a multi-ligand receptor by binding to the transmembrane protein, amnionless. We established a system to quantitatively analyse membrane targeting of the protein complex in cultured renal and intestinal cells and analysed the pathogenic mechanisms of mutations found in IGS patients. A novel *CUBN* mutation, several previously reported *CUBN* missense mutations and all previously reported *AMN* missense mutations resulted in endoplasmic reticulum (ER) retention and completely inhibited amnionless-dependent plasma membrane expression of cubilin. The ER retention of cubilin and amnionless was confirmed in renal proximal tubular cells of a patient with IGS. Notably, the interaction between cubilin and amnionless was not sufficient, but amnionless-mediated glycosylation of cubilin was necessary for their surface expression. Quantitative mass spectrometry and mutagenesis demonstrated that N-linked glycosylation of at least 4 residues of cubilin protein was required for its surface targeting. These results delineated the molecular mechanisms of membrane trafficking of cubilin in renal and intestinal cells.

## Introduction

Intestinal absorption of the intrinsic factor–B12 complex and the renal reabsorption of filtered low molecular proteins require multi-ligand receptor complexes formed by cubilin and amnionless^1–3^. Cubilin is a large protein with three types of domains, an N-terminal stretch, eight epidermal growth-factor (EGF)-like repeats and 27 CUB domains (defined as Complement C1r/C1s, Uegf and Bmp1)^4^. Cubilin binds to a variety of ligands and is crucial for renal tubular reabsorption of various proteins by glomerular ultrafiltration and for intestinal uptake of dietary vitamin B12 complexed with its transport protein intrinsic-factor^4,5^. Cubilin protein does not have a transmembrane region, and binding of its EGF-like repeats with a transmembrane protein, amnionless, enables its expression at the plasma membrane^6^. Cubam, a complex of cubilin and amnionless^7^, functions as a multi-ligand receptor complex and is expressed in a variety of tissues, including the kidneys, ileum and yolk sac^5,8,9^. In the kidney, megalin, a large glycosylated protein of 600 kDa sharing structural similarities with the endocytic receptors of the LDL receptor family, binds to cubilin and promotes its endocytosis and that of its ligands^2,3,10^.

Imerslund–Gräsbeck syndrome or juvenile megaloblastic anaemia 1 (IGS or MGA1; OMIM #261100) is an autosomal recessive disorder caused by mutations either the gene for amnionless (*AMN*) or that for cubilin (*CUBN*)^11,12^. Patients with IGS present with low molecular weight proteinuria and megaloblastic anaemia attributed to selective intestinal malabsorption of cobalamin. Cubilin deficiency caused by a homozygous truncating mutation of *CUBN* results in impaired apical expression of both cubilin and amnionless^13^. Conversely, in a spontaneous IGS canine model with an in-frame deletion of 33 nucleotides in an amnionless homologue, cubilin had an abnormal vesicular distribution in tubular cells^14^. This suggested an interdependent relationship between cubilin and amnionless. In cultured cells, formation of the cubam complex allows expression of mature glycosylated cubilin and cubilin is secreted at the apical surface in a glycosylation-dependent process^6^. To date, the effects of the missense mutations found in IGS patients on interactions of the cubam complex, interdependent membrane expression or endocytosis are not known.

We established a system to quantitatively assess membrane targeting of the protein complex in cultured renal and intestinal cells and analysed effects of a novel *CUBN* missense mutation and several other missense mutations of *CUBN* and *AMN* on their surface expression.

## Results

### Interdependent plasma membrane expression of cubilin and amnionless

**Figure 1:**
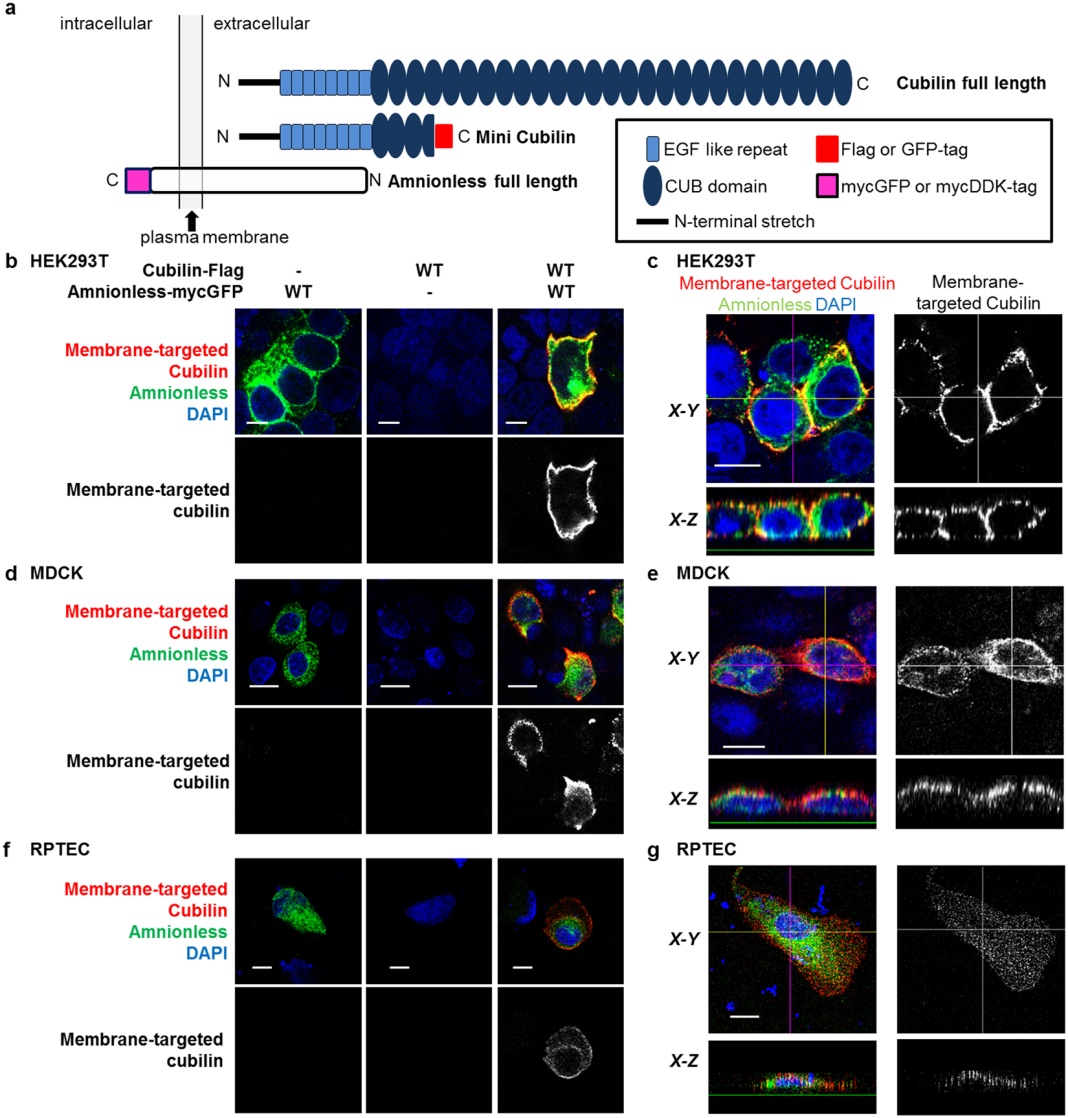
Interdependent membrane expression of cubilin and amnionless. (**a**) Rat cubilin (full-length and mini cubilin; 1–930) and human amnionless (full length) constructs encoded by plasmid cDNA used for transient transfection of cultured cells. (**b**,**d**,**f**) Non-permeabilised HEK293T, MDCK cells and RPTECs transfected with the indicated vectors were fixed and stained for membrane-targeted cubilin (red). GFP-tagged amnionless is shown in green and DAPI nuclear staining in blue. (Scale bar: 10 µm). (**c**,**e**,**g**) Plasma membrane expressions of cubilin were obtained in permeabilised HEK293T, MDCK cells and RPTECs cotransfected with amnionless. Pictures are confocal sections taken from the middle height of cells (*top*) and *X-Z* vertical sections (*bottom*) (Scale bar: 10 µm).

First, we analysed the molecular mechanism of membrane targeting of cubam using human embryonic kidney (HEK) 293 T cells, which do not endogenously express cubilin or amnionless (Supplementary Figure S1) but express an exogenous functional cubilin fraction including an N-terminus, eight EGF-like repeats and four CUB domains and amnionless (Fig. 1a). Expression of cubilin and amnionless was analysed in permeabilised cells (Supplementary Figure S2a). Flow cytometry results demonstrated that about 95% of amnionless-expressing cells also expressed cubilin when it was co-expressed (Supplementary Figure S2b).

Surface expression of cubilin and amnionless were analysed using non-permeabilised HEK293T cells (Fig. 1, and Supplementary Figure S3). When either cubilin or amnionless was expressed alone in cultured cells, membrane expression of the proteins was barely detected. However, when cubilin was co-expressed with amnionless, a fraction of cubilin expressed at the plasma membrane was detected (Fig. 1b–c). The interdependent membrane expression of cubilin and amnionless was also confirmed by analysis in Madin-Darby canine kidney (MDCK) cells (Fig. 1d and e, Supplementary Figure S3c), human primary renal proximal tubule epithelial cells (RPTEC) (Fig. 1f and g), and the human cultured colorectal cell line HCT116 (Supplementary Figures S3b and S4a). Among these cell lines, HEK293T cells and RPTECs expressed megalin (*LRP2*) mRNA, but HCT116 (Supplementary Figure S1) and MDCK cells^15^ did not. This indicated that amnionless-dependent plasma membrane targeting of cubilin did not require megalin expression.

### Mutations in *CUBN* and *AMN* caused defects in intracellular trafficking

**Figure 2:**
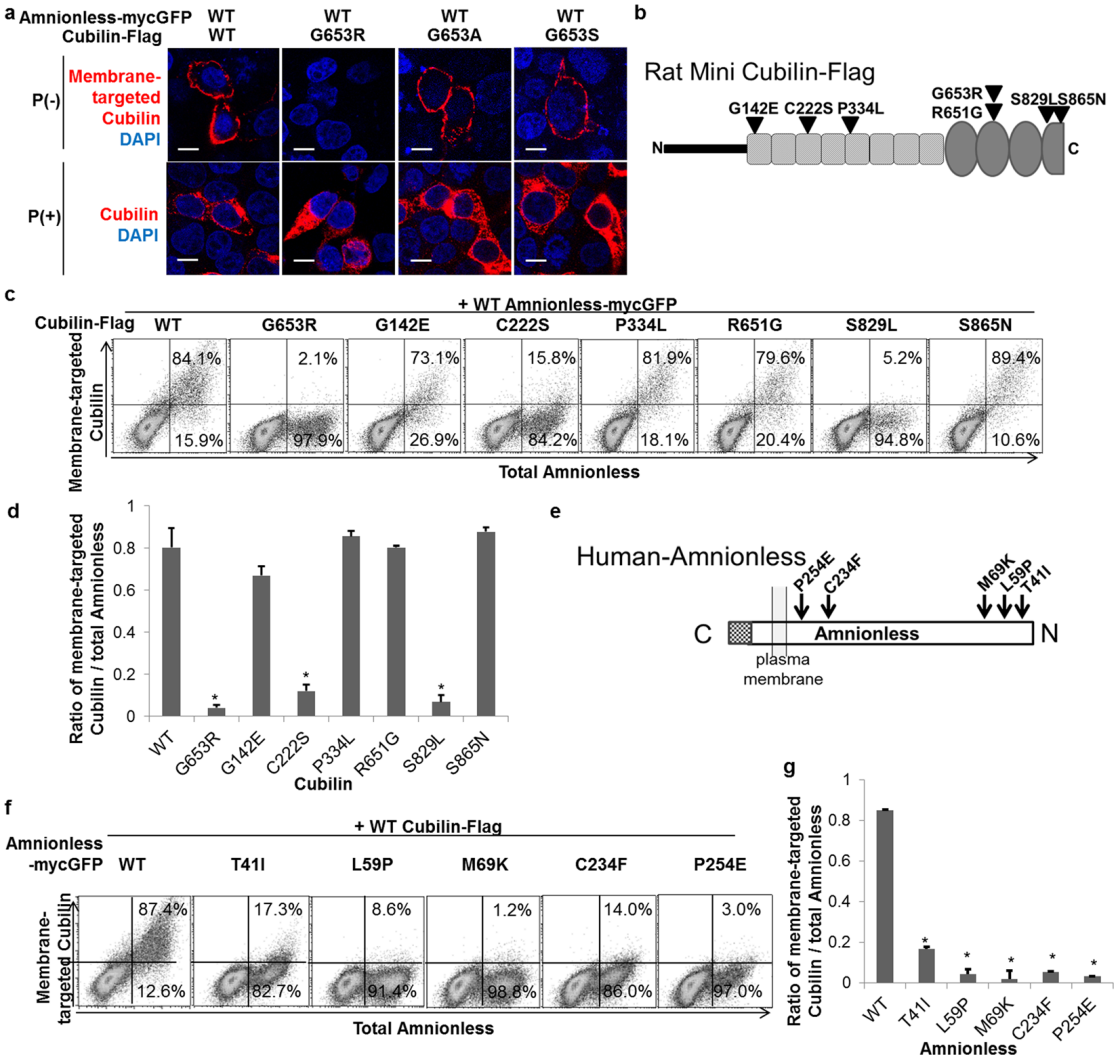
Missense mutations of cubilin and amnionless inhibit membrane expression of cubilin. (**a**) Non-permeabilised HEK293T cells transfected with indicated vectors were fixed and stained for membrane targeted cubilin. DAPI nuclear staining is shown in blue. (P: Permeabilisation, Scale bar: 10 µm) (**b**) Schematic representations of mutation sites in rat mini-cubilin. (**c**) Expression of GFP-tagged amnionless (x-axis) and membrane expression of cubilin (y-axis) was analysed by flow cytometry. (**d**) The ratio of cells with amnionless-dependent membrane-targeted cubilin to amnionless-expressing cells analysed by flow cytometry. Statistical data from three independent experiments are shown. Data represent means ± SEM. Statistical significance: *P < 0.01. (**e**) Schematic representations of mutation sites in human amnionless. (**f**) Cubilin-Flag was co-expressed with WT or mutant amnionless-mycGFP in HEK293T cells. Membrane expression of amnionless was analysed by flow cytometry. (**g**) The ratio of cells with amnionless-dependent membrane-targeted cubilin to amnionless-expressing cells was analysed by flow cytometry. Statistical data from three independent experiments are shown. Data represent means ± SEM. Statistical significance: *P < 0.01.

We next examined the effects of the *CUBN* mutations found in IGS patients, including a novel G653R mutation in a 6-year-old Japanese male with megaloblastic anaemia and low-molecular weight proteinuria (Supplementary information, Supplementary Figure S5), on membrane expression of cubam. The *CUBN* G653R mutation dramatically decreased amnionless-dependent cubilin membrane expression (Fig. 2a) in HEK293T cells. On the other hand, reported polymorphisms in G653 (G653A, and G653S) (Supplementary Table 1a) did not affect membrane targeting of cubilin (Fig. 2a). Defective membrane targeting by the G653R mutation was also observed in HCT116 cells, MDCK cells and RPTECs (Supplementary Figure S4). This effect was dose-dependent because membrane expression of cubilin was partially inhibited when wildtype and G653R cubilins were co-expressed (Supplementary Figure S6), indicating that the G653R mutation is a loss-of-function mutation. The G653R mutation also inhibited cubilin-dependent amnionless membrane expression in HEK293T, HCT116 and MDCK cells (Supplementary Figure S3).

Next, to analyse whether the G653R mutation abrogated plasma membrane targeting of cubilin or accelerated its endocytosis, we examined the effects of the mutation on cubilin internalisation^6^. As shown in Supplementary Figure S7, cells double-transfected with wildtype cubilin and amnionless exhibited strong vesicular labelling, indicating that mini-cubilin was internalised from the apical surface, whereas cells that only expressed cubilin did not internalise the anti-Flag antibody. The vesicular signal was not observed in cubilin G653R mutants. These data demonstrated that decreased membrane expression was not caused by increased cubilin uptake from the cell membrane but, instead, by decreased membrane targeting.

Among the previously reported six mutations in N-terminus cubilin (Fig. 2b, Supplementary Table 1)^16,17^, C222S and S829L had defects in cubilin membrane expression (Fig. 2c and d). Notably, all previously reported *AMN* mutations (T41I, L59P, M69K, C234F and P254E, Fig. 2e, Supplementary Table 2)^16,18^ had defects in amnionless-dependent membrane targeting of cubilin (Fig. 2f and g).

**Figure 3:**
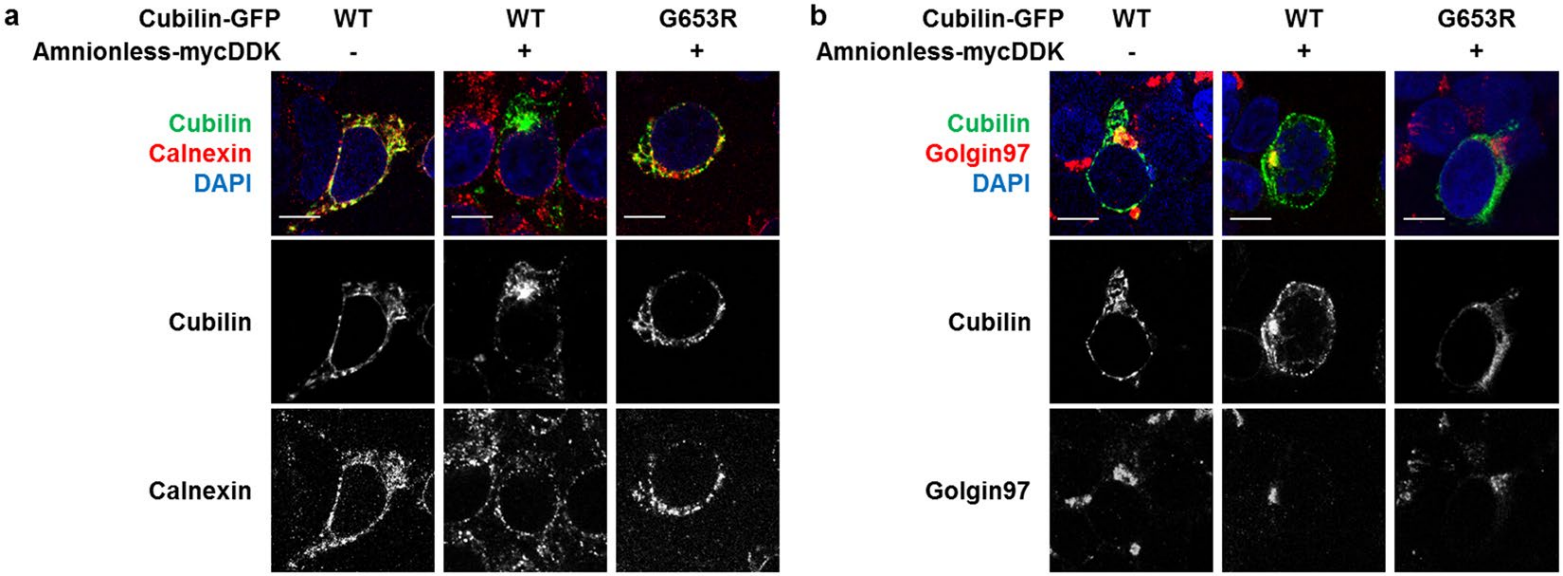
Cubilin mutation inhibits its trafficking from endoplasmic reticulum to Golgi apparatus. (**a** and **b**) HEK293T cells transfected with indicated vector were fixed, permeabilised and labeled for calnexin (**a**), the endoplasmic reticulum marker, or golgin 97 (**b**), a marker of the Golgi apparatus, followed by Alexa Fluor 555-conjugated antibody (red). GFP-tagged cubilin is shown in green and DAPI nuclear staining in blue. Pictures are confocal sections taken from the middle height of cells. (Scale bar: 10 µm).

Regarding intracellular localisation, cubilin was retained in the endoplasmic reticulum (ER) when expressed in cultured cells (Fig. 3a). However, in cells co-expressing cubilin and amnionless, a small but distinct fraction of membrane-targeted cubilin was detected (Figs 1 and 3). Importantly, intracellular localisation of cubilin was changed from the ER to the Golgi apparatus by co-expression with amnionless in HEK293T cells (Fig. 3). Cubilin translocation to the Golgi apparatus by amnionless was also confirmed in transfected MDCK cells (Supplementary Figure S8). The *CUBN* G653R mutation resulted in retention of cubilin in the ER even when co-expressed with amnionless (Fig. 3, Supplementary Figure S8). These findings suggested that the pathogenic mutation caused the ER accumulation, which led to defective expression at the cell surface.

### Retention of cubilin in the ER in renal tubules of an IGS patient

**Figure 4:**
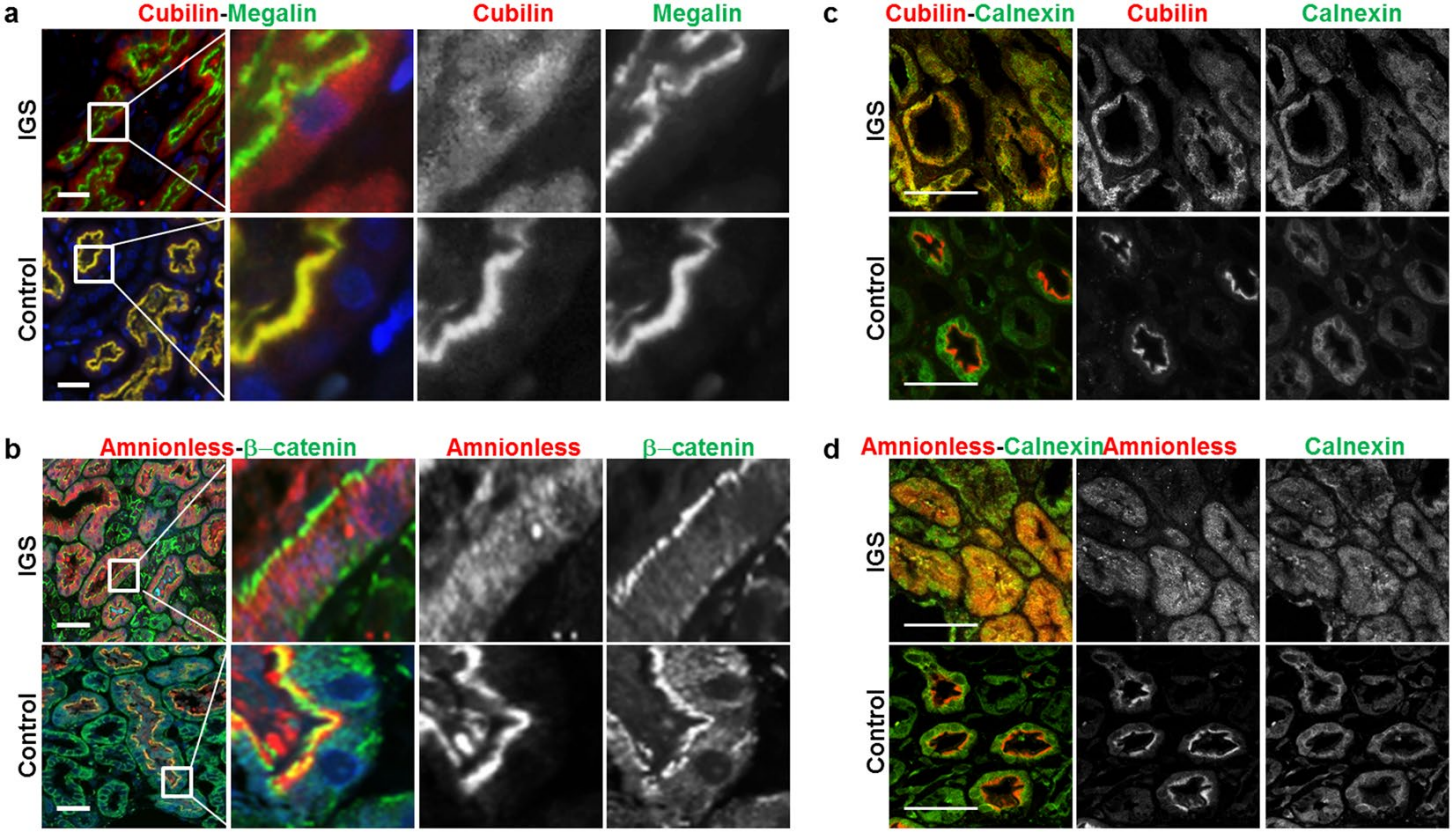
Expression of cubilin and amnionless in human kidney cortex. Multi-labeling immunofluorescence staining for cubilin (red) and megalin (green) (**a**) or amnionless (red) and β-catenin (green) (**b**) in deparaffinized-embedded renal sections from the IGS patient and from a control subject (9-year-old boy with minimal-change nephrotic syndrome). Megalin is a multi-ligand binding receptor expressed in the apical membrane of tubular cells. β-catenin is expressed in the apical membrane of proximal tubular cell^34^. (**c** and **d**) While cubilin and amnionless were localized at the apical surface of proximal tubular cells in the control patient, those in the IGS patient showed abnormal intracellular localization. (Scale bar: 50 µm) Multi-labeling immunofluorescence staining for calnexin (green) and cubilin (red, (**c**)) or amnionless (red, (**d**)) in deparaffinized-embedded renal sections from the IGS patient and from a control subject. Cubilin and amnionless in the IGS patient were localized with calnexin. (Scale bar: 50 µm).

By light microscopy, a renal biopsy sample from a patient with the *CUBN* G653R mutation revealed normal glomeruli and focal interstitial fibrosis and tubular atrophy (Supplementary Figure S5c). As previously reported in a healthy subject^13^, amnionless and cubilin expression were detected along the brush borders of proximal tubular cells in a specimen of a control subject (Fig. 4a and b). However, in the IGS patient, expression of cubilin and amnionless was observed in the ER of proximal tubular cells (Fig. 4c and d).

### The cubilin–amnionless interaction is not sufficient for membrane targeting

**Figure 5:**
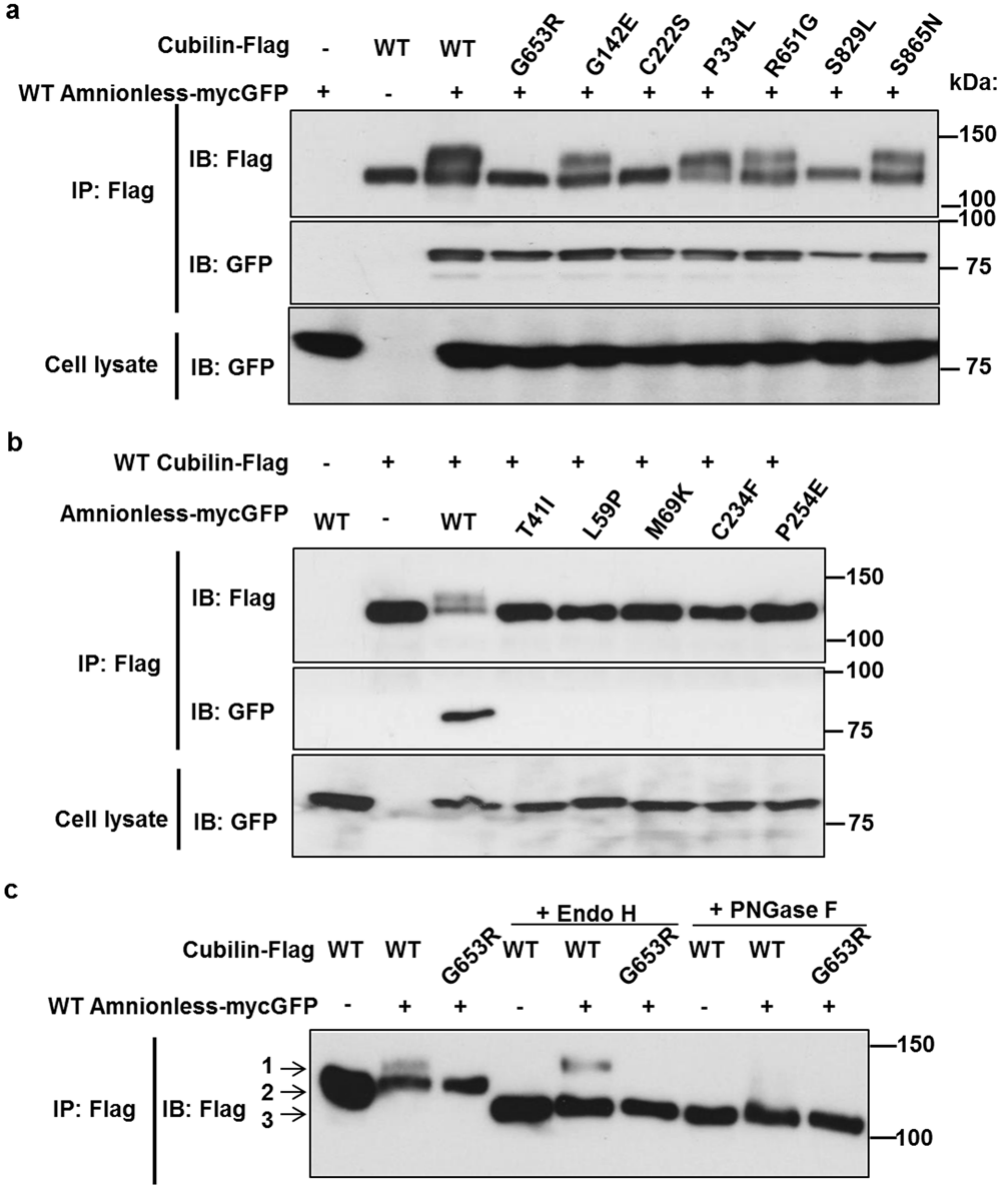
Missense mutations of *CUBN* and *AMN* abrogated amnionless-dependent N-glycosylation of cubilin. (**a**–**c**) HEK293T cells were transfected with the indicated vectors. Anti-Flag immunoprecipitates and cell lysates were analysed by western blotting with the anti-Flag antibody for cubilin-flag and anti-GFP antibody for amnionless-mycGFP. WT: wild-type; IP: Immunoprecipitation; IB: immunoblotting. Full-length blots are presented in Supplementary Figure 12. (**a**) Missense mutations of *CUBN* G653R, C222S, and S829L inhibited amnionless-dependent N-glycosylation of cubilin. (**b**) All the previously reported missense mutations of *AMN* inhibited amnionless-dependent N-glycosylation of cubilin. Note that all these mutations caused complete defects in its interaction with cubilin. (**c**) Anti-Flag immunoprecipitates were treated with endoglycosidase H (Endo H) or peptide-N-glycosidase F (PNGase F), and analysed by western blotting. Arrows point to the mature form (1), the immature form (2), and the deglycosylated form (3).

Our next question was whether defects in membrane targeting of cubam were caused by defects in the interaction between cubilin and amnionless. Interaction of wildtype cubilin and amnionless was confirmed by immunoprecipitation (Fig. 5a) and a proximity ligation assay (Supplementary Figure S9). Immunoprecipitation analysis demonstrated that, although all the amnionless mutations found in IGS patients caused defective binding with cubilin (Fig. 5b), this interaction was not affected by cubilin mutations (Fig. 5a). This indicated that some factor other than cubam interaction was crucial for interdependent plasma membrane targeting.

### IGS mutations caused defects in amnionless-dependent glycosylation of cubilin

Formation of the cubilin–amnionless complex was shown to promote cubilin maturation^6^. Cubilin undergoes N-glycosylation, resulting from the attachment of a sugar moiety to an asparagine (Asn, N) in the consensus sequence: N–X–S/T. Tunicamycin-sensitive N-glycosylation of cubilin was shown to important for apical sorting of cubilin–amnionless complexes^6^. In cells expressing cubilin alone, cubilin was glycosylated in an Endo-H sensitive form (Fig. 5c, arrow “2”), indicating that its glycosylation had not yet undergone full maturation. Together with the immunofluorescence findings (Fig. 3), when cubilin was expressed alone, N-linked sugars were added in the ER but the proteins did not appear to traffic to the Golgi complex.

Co-expression of amnionless and cubilin resulted in the appearance of additional higher–molecular weight forms of cubilin (Fig. 5c, arrow “1”). The more slowly migrating proteins were sensitive to treatment with PNGase F, which removes all N-linked sugars, but were resistant to treatment with Endo H. This indicated that this fraction of cubilin had reached the medial Golgi complex.

The amnionless-dependent stepwise glycosylation of cubilin was abrogated in the *CUBN* G653R, C222S and S829L mutations (Fig. 5a) and all the *AMN* missense mutations reported previously (Fig. 5b).

### Identification of glycosylation sites in mini-cubilin

**Figure 6:**
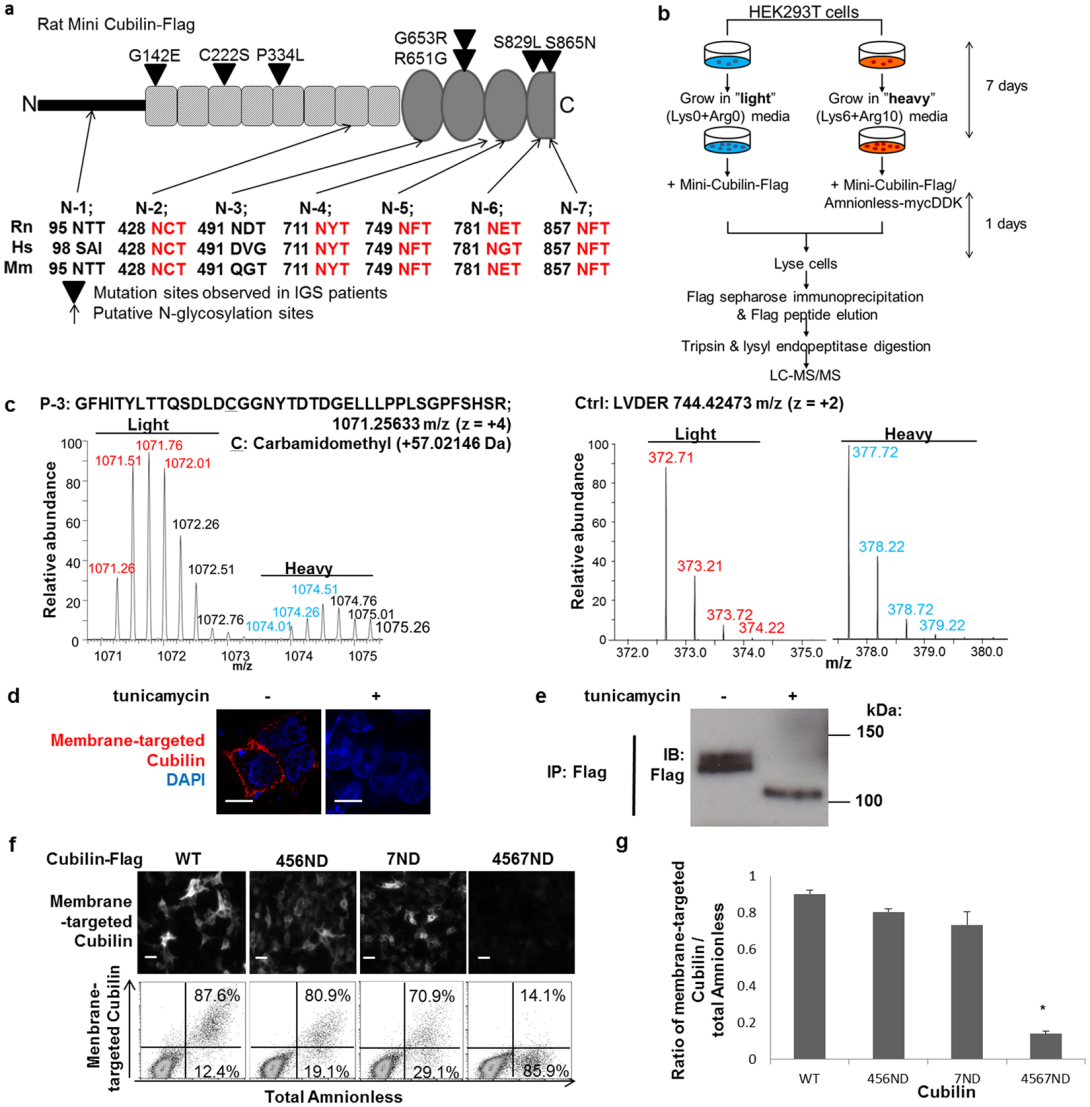
Glycosylation defect of cubilin abrogated amnionless-dependent membrane expression of cubilin. (**a**) Schematic representations of N-glycosylation sites in mini-cubilin. The conservation across multiple species of consensus amino acid sequences for N-glycosylation sites is shown. Hs: *Homo sapiens*; Rn: *Rattus norvegicus*; Mm: *Mus musculus*. (**b**) A schematic diagram of SILAC labeling and proteome analysis. HEK293T cells were labeled with light (L-^12^C_6_^14^N_4_-Arg and L-^12^C_6_-Lys) or heavy (L-^13^C_6_^15^N_4_-Arg and L-^13^C_6_-Lys) isotope-labeled amino acids. Cells transiently transfected with wild-typed cubilin-Flag only or wild-type cubilin-Flag and amnionless-mycDDK were cultured in ‘Light’ or ‘Heavy’ medium, respectively. Equal amounts of cell lysate from ‘Light’ and ‘Heavy’ cells were mixed and immunoprecipitated with anti-Flag antibody. After trypsin/lysyl endopeptidase digestion, peptides were analysed by LC-MS/MS. (**c**) Representative spectral data of cubilin peptides including N-glycosylation site (P-3) or control not including N-glycosylation site using SILAC. (**d**) HEK293T cells transfected with both wild-type cubilin-flag and wild-type amnionless-mycGFP were incubated in absence or presence of tunicamycin (4 mg/mL) for 18 h at 6 h after transfection. Non-permeabilised cells were stained for membrane-targeted cubilin (red). (Scale bar: 10 µm.) (**e**) Expression levels of cubilin-flag in (**d**) were analysed by immunoblotting. Anti-flag immunoprecipitates were separated on a 7.5% SDS-PAGE gel, followed by western blotting. Full-length blots are presented in Supplementary Figure 13 (**f**) Non-permeabilised HEK293T cells transfected with amnionless-mycGFP and cubilin-Flag (wild-type or Asn to Asp (ND) mutants) were stained for membrane-targeted cubilin. Plasmid constructions of the ND mutant were created by replacement of Asn for Asp in the potential N-glycosylation sites as shown in Fig. 6a. Cubilin 7ND, replacing N857 with D857; Cubilin 456ND, replacing a combination of N711, N749, and N781 with a combination of D711, D749, and D781; Cubilin 4567ND, replacing N857 with D857 added to Cubilin 456ND. Membrane expression of cubilin and amnionless analysed by flow cytometry. (**g**) The ratio of cells with amnionless-dependent membrane-targeted cubilin to amnionless-expressing cells was analysed by flow cytometry in (**f**). Data represent means ± SEM. Statistical significance: *P < 0.01.

Analysis of the primary sequence of rat mini-cubilin using the NetNGlyc1.0 prediction server (Center for Biological Sequence Analysis, Technical University of Denmark) revealed the presence of seven potential N-glycosylation consensus sites at positions described as N-1 to N-7 (Fig. 6a). N-1 and N-2 are located in the N-terminal stretch and EGF-like repeats, respectively and the rest are located in the CUB domains. Among them, N-2, N-4, N-5, N-6, and N-7 are conserved in human, mouse and rat cubilins.

To quantify the relative amounts of modification at each potential N-glycosylation consensus site of cubilin, mass spectrometric analysis was performed. The quantification was performed using stable isotope labelling by amino acids in cell culture (SILAC) technology (Fig. 6b). Mini-cubilin was expressed in HEK293T cells with amnionless in medium containing heavy-labelled amino acids or without amnionless in a medium containing light-labelled amino acids. The peptides generated from cubilin protein, with and without amnionless, were mixed and subjected to nano-LC-MS/MS analysis. Among six peptides containing candidate glycosylated Asn, three peptides, P-1 (containing N-2), P-3 (containing N-4) and P-5 (containing N-6) were identified and their relative amounts (heavy/light) were calculated (Table 1). Among the three peptides, P-3 had a lower heavy/light ratio (Fig. 6c) than did most of the other peptides derived from mini-cubilin, suggesting that the amount of post-translational modification, most likely glycosylation, at P-3 peptides was increased in the presence of co-expressed amnionless. A similar change in the mass of P-3 peptide was confirmed in cells transiently transfected with G653R or wildtype cubilin–Flag and amnionless–mycDDK cultured in ‘light (L)’ or ‘heavy (H)’ medium, respectively (Supplementary Figure S10).

**Table 1:** Proteomic identification of cubilin peptide with amnionless-dependent post-translational modification. HEK293T cells were labeled with light (L-^12^C_6_^14^N_4_-Arg and L-^12^C_6_-Lys) or heavy (L-^13^C_6_^15^N_4_-Arg and L-^13^C_6_-Lys) isotope-labeled amino acids. Cells transiently transfected with wild-typed cubilin-Flag or with both cubilin-Flag and amnionless-mycDDK were cultured in ‘light (L)’ or ‘heavy (H)’ medium, respectively. Equal amounts of cell lysate from ‘light’ and ‘heavy’ cells were mixed and immunoprecipitated with anti-Flag antibody. After trypsin/lysyl endopeptidase digestion, peptides were analysed by LC-MS/MS (Fig. 6b). Spectral data of each paired cubilin-peptide (e.g. Fig. 6c) were used to calculate the ratios (H/L) that give the quantitative response of change in peptide mass by co-expression with amnionless. In each experiment, the average of high-to-light ratios of cubilin peptides excluding six peptides with possible glycosylation (P1-P6) was used as a standard (ratio: 1) for adjusting the efficacy of amino acid replacement. Asn (N) residues are highlighted by underlined characters. N/D: not detected; Exp.: Experiment.

Peptide No.	Sequence of peptides with predicted glycosylation sites in mini cubilin	Charge	m/z [Da]	MH + [Da]	Ratio of heavy/light		
					Exp. 1	Exp. 2	Exp. 3
P-1	N^95^TTGLPQNILSQVHQLNSK	3	698.043	2092.114	0.969	0.740	0.736
P-2	CDSGWSGQN^428^CTENINDCLSNPCLNGGTCIDGIN^491^FTCDCTSSWTGYYCQTPQAACGGILSGTQGTFAYHSPNDTYIHNVNCFWIVR	N/D	N/D	9227.029	N/D	N/D	N/D
P-3	GFHITYLTTQSDLDCGGN^711^YTDTDGELLLPPLSGPFSHSR	4	1071.256	4281.998	0.422	0.195	0.439
P-4	QCVYLITQAQGEQIVIN^749^FTHVELESQMGCSHTYIEVGDHDSLLR	N/D	N/D	4988.545	N/D	N/D	N/D
P-5	ICGN^781^ETLFPIR	2	660.341	1319.674	0.978	0.713	0.984
P-6	QVVLLN^857^FTDFQIGSSASCDTDYIEIGPSSVLGSPGNEK	N/D	N/D	3971.312	N/D	N/D	N/D
ctrl	LVDER	2	372.213	744.4273	1.197	1.040	1.129

Furthermore, the SILAC analysis using cells transiently transfected with G653R or wildtype cubilin–Flag and amnionless–mycDDK cultured in ‘light (L)’ or ‘heavy (H)’ medium, respectively, demonstrated that the complex of G653R cubilin and amnionless, but not that of wildtype cubilin and amnionless, binds to UDP-glucose: glycoprotein glucosyltransferase 1 (Uggt1) (ratio of heavy/light: 0.098).

### Specific N-glycosylation of cubilin regulated the surface delivery of cubam

Finally, the effect of cubilin glycosylation on its membrane expression was analysed. Tunicamycin, an inhibitor of N-glycosylation in the ER, completely inhibited amnionless-dependent cubilin membrane targeting (Fig. 6d and e). Because substitution of each seven single Asn residues with Asp did not affect membrane targeting of cubilin (data not shown), we designed a series of mutants combining glycosylation sites to probe the functional ramifications of changes at multiple sites in concert. While combined ND (Asn to Asp) mutations in N-4, N-5 and N-6 (456ND) or a single ND mutation in N-7 (7ND) did not inhibit cubilin membrane targeting, combined ND mutations in N-4, N-5, N-6 and N-7 (4567ND) almost completely blocked cell surface expression of cubilin (Fig. 6f and g). The combined substitution (4567ND) did not affect cubam complex formation (Supplementary Figure S11). Notably, the 4567ND mutation, but not the 7D or 456ND mutations, completely inhibited its mature glycosylation (Supplementary Figure S11). These results demonstrated that specific combined N-glycosylation in the CUB domains was essential for maturation of cubam complexes and their surface localisation.

## Discussion

Genetic or other alterations in the pathways of membrane transport have severe functional consequences and are associated with several human diseases^19^. In this study, we delineated the mechanisms of intracellular trafficking of cubilin and amnionless, showing that mutations of *CUBN* and *AMN* cause ER retention, glycosylation and abrogation of surface expression.

Some previously reported *CUBN* mutations (G145E, P337L, R651G and S865N) did not inhibit interaction with amnionless or surface expression of cubam in our *in vitro* system. Among them, the heterozygous R651G mutation was found in two siblings of a family in Taiwan^16^. *CUBN* R651 is conserved among vertebrates and the mutation is considered “Probably Damaging” by PolyPhen-2. However, the allele frequency of the variant, rs182512508, is 0.01658 in the East Asian population in gnomAD^20^ (Supplementary Table 1), which seems too high to be a pathogenic mutation for this rare disorder. *CUBN* S865 is not conserved among mammals and S865N (rs138083522, Allele frequency 0.007404) is considered a “Benign” amino acid change in PolyPhen-2. Notably, *CUBN* S865N was reported in a homozygous state in six individuals in the cohort of the gnomAD^20^. Together with the results of our study, this suggested that these variations have no functional relevance. The potential mechanisms underlying the malfunction of the cubam complex with mutations that do not inhibit cubilin glycosylation (G145E and P337L) include glycosylation defects in other parts of the cubilin molecule that we did not analyse in our study, or defects in binding with ligands, endocytosis or receptor recycling with intact surface expression.

N-glycosylation is a major post-translational modification regulating protein folding, stability, intercellular trafficking and protein complex formation^21^. Cubilin has 42 predicted N-glycosylation sites and we identified candidate amnionless-dependent glycosylation sites of cubilin using peptide mass analysis. Mutation analysis revealed that addition of a combination of several N-glycans in the CUB domain was required for the trafficking of both cubilin and amnionless. Notably, the *CUBN* C222S, G653R, and S829L mutations led to disturbances in cubilin glycosylation without affecting the interaction between cubilin and amnionless, which requires the N-terminal EGF-like repeats of cubilin (amino acids 107–452)^6^. G653 and S829 are in CUB domains and are located at a distance from the binding domain; therefore, the interaction would not be affected by G653R and S829L. However, C222S in the EGF-like domain did not interfere with the interaction between amnionless and cubilin, but abrogated cubilin glycosylation status (Fig. 5a). These results led us to surmise that an intact higher-order structure of cubilin, but not only an interaction, was crucial for export from the ER and mature glycosylation, which are, in turn, crucial for its surface expression.

During protein folding in the ER, newly synthesised N-linked glycoproteins bind to calnexin (Cnx)/calreticulin (Crt) immediately upon their entry into the lumen of the ER as it moves toward its proper structure. Uggt1 recognises the incompletely folded glycoprotein and catalyses readdition of a glucosyl residue to the high-mannose oligosaccharides to facilitate another round in the Cnx/Crt folding cycle^22,23^. The observation that G653R cubilin, but not wildtype cubilin, bound to Uggt1 when co-expressed with amnionless, demonstrated that the cubam complex formed by mutant cubilin did not pass the checkpoint for mis-folded proteins.

Similar mechanisms employed by other membrane protein complexes serve as useful models for examining the process of membrane targeting and receptor function. For example, dystroglycan is a heterodimer comprising a transmembrane β-subunit and a large, heavily glycosylated α-subunit^24,25^. α-Dystroglycan and β-dystroglycan associate within the protein export pathways and this protein complex undergoes complex glycosylation. Mutations in these genes or in genes involved in post-translational modification of the dystroglycan complex were shown to cause multiple forms of recessive muscular dystrophy^25^. Another example is the adenosine triphosphate-binding cassette (ABC) transporter G5/G8 expressed in the liver and small intestine, critical for protecting the body from accumulating dietary plant sterols. ABCG5 and ABCG8 form a heterodimer in the ER and are N-glycosylated prior to being transported to apical membranes^26^. Our results suggested that the cubam complex employs a similar mechanism of assembly and trafficking to the cell membrane. In other proteins requiring heterodimerisation to exit the ER^26–29^, several ER retention motifs are known to be masked by heterodimerisation, enabling the protein complex to exit the ER. The details of protein sorting motifs in cubilin or amnionless proteins are unclear. Additional studies are required to determine the molecular mechanisms by which amnionless and cubilin are recognised by the ER quality control system and the effects of mutations found in IGS patients. The mechanism of ER retention of cubam complexes will serve as a potential therapeutic target for IGS.

The results of our study provided evidence that the de novo *CUBN* G653R mutation was pathogenic. In the patient we analysed, a candidate mutation in exon 14 of *AMN* (c.883 C > T (p.P295S)), which was inherited from the patient’s mother, was also found. The mutation was not registered as a SNP in the dbSNP, ExAC or Japanese SNP databases. PolyPhen-2 and SIFT predicted that mutation is “Benign” and “Tolerated”, respectively and the result of the likelihood ratio test was “Unknown”. Effects of the *AMN* P295S mutation on amnionless-dependent cubilin membrane expression or glycosylation were not as significant as those of other *AMN* mutations (data not shown). Therefore, the contribution of the *AMN* P295S mutation to pathogenesis was inconclusive, although it was still possible that the mutation caused additive defects in cubam membrane expression leading to a loss of functional cubilin that was sufficient to cause IGS.

Histopathological analysis of specimens from an IGS patient with a *CUBN* splice donor site mutation^13^, the IGS patient in our study (Fig. 4) and a transgenic cubilin knockout mouse^10^ showed that megalin distribution was unaffected by cubilin expression. Conversely, we demonstrated that amnionless-mediated plasma membrane targeting of cubilin did not require endogenous megalin expression (Supplementary Figures S1 and S4). These results indicated that surface expression of cubilin and megalin were mainly independent. Functionally, it was reported that megalin increased uptake of intrinsic factor–vitamin B12 complex, mediated by cubilin–amnionless complexes, and that the main role of megalin in albumin reabsorption is to drive the internalisation of cubilin-amnionless complexes^10^. The mechanistic details of anchorage, processing and recycling of the cubilin–amnionless–megalin complex are important issues for future investigation.

*CUBN* gene variation is not implicated only in IGS. Recently, genome-wide association study analysis of a general population identified a missense variant in the *CUBN* gene as a risk allele for a high urinary albumin-to-creatinine ratio and micro albuminuria^30^. While the effects of this variant on membrane targeting or endocytosis of cubam are unknown, our results raised the possibility that the defective intracellular trafficking of cubilin may not only cause IGS, but also be related to more general pathologic conditions.

In summary, mutations of *CUBN* and *AMN* in IGS patients caused complete defects in export from the ER, mature glycosylation and membrane targeting of cubam, highlighting the importance of post-translational modifications for biogenesis and stability of the complex. Our results suggested that defective intracellular trafficking of cubilin is the mechanism underlying impairments in renal and intestinal absorption in IGS.

## Methods

### Antibodies

The following antibodies were obtained commercially: rabbit polyclonal anti-amnionless antibody (1: 250, HPA000817; Sigma-Aldrich, St. Louis, MO), goat polyclonal anti-cubilin antibody (1: 50, T-16; Santa Cruz Biotechnology, Dallas, TX), mouse polyclonal anti-β catenin antibody (1: 200, R&D Systems, Minneapolis, MN) mouse monoclonal anti-Flag antibody (1: 2000, M2; Sigma-Aldrich), mouse monoclonal anti-GFP antibody (1: 2000, B-2; Santa Cruz Biotechnology), mouse monoclonal anti-calnexin antibody (1: 50, E-10; Santa Cruz Biotechnology), and mouse monoclonal anti-golgin 97 antibody (1: 100, A-21270; Invitrogen, Carlsbad, CA). Rabbit polyclonal anti-megalin antibody was previously described (1: 200)^31^.

### Immunohistological analysis

Tissues for light microscopy were collected with informed consent from all subjects and processed routinely. Paraffin-embedded samples from human renal biopsy samples were deparaffinized in xylene and rehydrated through a series of graded alcohols in H_2_O, followed by heat-induced epitope retrieval by incubating in a target retrieval solution (S1699; Dako, Carpinteria, CA) for 15 min at 121 °C. Sections were cooled to room temperature and incubated with the primary antibodies, followed by incubation with secondary antibodies (Invitrogen). Confocal fluorescent images were obtained using a Zeiss LSM 700 confocal microscope. Images were processed using Adobe Photoshop CS6.

### Expression vectors

Plasmids containing full length cDNA for wild-type human amnionless with the C-terminal mycDDK tag were purchased from OriGene Technologies (Rockville, MD). The mini-cubilin plasmid coding region of rat cubilin including 1–2784 bp cDNA (1–928AA) was amplified by PCR using the following specific primer sets: 5′-GGAATAAGCTTGCCATGTCCTCGCAGTTTCTCTGGGG-3′ and 5′-CACGTCTCGAGGTCACTGCTGAACTTAGCAC-3′, and cloned into the appropriate cloning site of the pCMV4A vector (wild-type rat cubilin-Flag). The eGFP was amplified from a pEGFP-N1 vector using primers with an XhoI site in the forward primer and an ApaI site and putative splice acceptor site in the reverse primer. Rat cubilin-Flag and the amplified eGFP were digested with XhoI and ApaI (and isoschizomer of EcoRV). Fragments were ligated to create pCMV-wild-type rat cubilin-GFP. Rat cubilin-GFP and human amnionless-mycDDK were digested with BamHI and EcoRV (and isoschizomer of EcoRI). Fragments were ligated to create pCMV-human amnionless-mycGFP. Plasmids expressing cubilin or amnionless mutants were generated by PCR-based mutagenesis using wild-type cubilin or wild-type amnionless plasmids as the template.

### Cell culture

HEK293T cells (ATCC, Manassas,VA), Madin-Darby canine kidney (MDCK) cells, Cos1 cells, HCT116 (ATCC) cells and Hela cells were grown in Dulbecco’s modified Eagle’s medium(DMEM) with 10% fetal bovine serum (FBS), sodium pyruvate, and penicillin-streptomycin. Human Renal Proximal Tubular Epithelial Cells (RPTECs: CC2553) (Lonza, Japan) were cultured in renal epithelial cell growth medium supplemented with 0.5% FBS. Cells were grown in a humidified atmosphere with 5% CO_2_ and 95% air at 37 °C.

### Transfection and co-immunoprecipitation

Plasmids were transfected into HEK293T or HCT116 cells using PEI-MAX (Polysciences Inc., Warrington, PA). RPTECs were transfected with Lipofectamine LTX and Plus Reagent (Invitrogen) and MDCK cells were transfected with Lipofectamine 2000 (Invitrogen). Cells were lysed in a lysis buffer (20 mM Tris-HCl (pH 7.5), 150 mM NaCl, 1 mM EDTA, and 1% NP-40) containing a protease inhibitor cocktail (Roche, Germany) for 15 min on ice. Lysates were clarified by centrifugation and incubated with agarose beads conjugated with anti-Flag antibody for 1 h at 4 °C. Beads were washed three times with lysis buffer and bound proteins were eluted with SDS sample buffer.

### Deglycosylation analysis

Cell lysates were resuspended in endoglycosidase-H (New England Biolabs, Ipswich, MA) or PNGase F (New England Biolabs) digestion buffer and processed following the manufacturer’s protocol. For analysis of inhibition of N-glycosylation *in vivo*, cells were incubated for 18 h with 4 µg/mL tunicamycin (Wako Pure Chemical Industries, Osaka, Japan).

### Immunostaining

Cells expressing cubilin, amnionless, or both were cultured on glass slides in 24-well plates (Falcon; BD Biosciences, San Jose, CA) under the conditions described above. For analysis of surface expression of cubilin, cells were fixed with 2% paraformaldehyde in phosphate-buffered saline (PBS) for 5 min. For analysis of protein expression, cells were fixed for 5 min and permeabilised by 0.1% Triton X-100 in PBS for 10 min. They were incubated with PBS with 1% skim milk for 30 min, followed with primary antibody for 1 h. Alexa Fluor 555 or 488 conjugated antibodies (1: 1000, Invitrogen) was used as a secondary detection antibody. Slides were mounted in a medium with DAPI (Life Technology, Carlsbad, CA). Stained cells were visualized using confocal microscope (LV300; Olympus, Tokyo, Japan), or a Zeiss LSM 700 confocal microscope (Fig. 1). Images were processed using Adobe Photoshop CS6.

### Flow cytometry

Cell surface expression of cubilin-Flag or amnionless-GFP was analysed by flow cytometry. Transfected HEK293T cells expressing cubilin and amnionless were fixed with 2% paraformaldehyde in PBS for 5 min and incubated with an anti-Flag antibody (for cubilin) or anti-amnionless antibody and an Alexa Fluor 647-conjugated secondary antibody (1:1000, Invitrogen). Stained-cells gating and data collecting were performed with FACS Calibur (BD Biosciences). Data were analysed using Flow Jo software (Takara Tomy, Japan).

### SILAC labeling

For SILAC labeling, cells were divided into two populations: one grown in SILAC DMEM medium supplemented with L-^12^C_6_^14^N_4_-Arg and L-^12^C_6_-Lys (light medium containing natural isotopes) and the other in SILAC medium supplemented with L-^13^C_6_^15^N_4_-Arg and L-^13^C_6_-Lys (heavy medium containing stable isotopes) (Thermo scientific, Meridian RD., Rockford). Both light and heavy media were supplemented with 10% dialyzed fetal bovine serum and 1% antibiotics. Cells were cultured in SILAC medium for at least three passages to achieve maximum labeling before examination.

### Analysis by mass spectrometry

Enzymatic digestion of protein samples were performed based on PTS method as reported previously^32^. In brief, samples dissolved in PTS buffer (12 mM sodium deoxy cholate, 12 mM sodium N-lauroylsarcosinate, 50 mM NH_4_HCO_3_) were reduced (10 mM dithiothreitol at RT for 30 min) and alkylated (50 mM 2-iodoacetamide at RT for 30 min), and then diluted to 5 folds by adding 50 mM NH_4_HCO_3_ solution. The samples were then digested with 1 μg Lysyl Endopeptidase (LysC; Wako Pure Chemical Industries, Osaka, Japan) for overnight, followed by further digestion with with 1 μg trypsin for 6 hr at room temperature. The digested products were mixed with equal volume of ethyl acetate containing 0.5% TFA allowing the detergents to dissolve into an organic phase. After the centrifugation at 10,000 × g for 10 min, an aqueous phase containing the peptides were recovered, and then dried with SpeedVac (Thermo Fisher Scientific). The samples were dissolved in 2% acetonitrile with 0.1% TFA and then desalted by using self-prepared STAGE tips^33^. Samples were stored at −80 °C and dissolved in 2% acetonitrile with 0.1% TFA before analysis by mass spectrometer.

The mass spectrometry analysis was carried out by data-dependent MS/MS with a Q-FT mass spectrometer (Q-Exactive, Thermo Fisher Scientific) equipped with a nano HPLC system (Advance UHPLC, Bruker Daltonics) and an HTC-PAL autosampler (CTC Analytics) with a trap column (0.3 × 5 mm, L-column, ODS, Chemicals Evaluation and Research Institute, Japan). The sample was loaded and separated by nano HPLC with a gradient mixture of mobile phase A (0.5% acetic acid) and B (0.5% acetic acid and 80% acetonitrile) at a flow late 300 nL/min and analysed by nanoscale micro-capillary LC-MS/MS. The Supplementary gradient was either of following conditions: (1) 4% to 36% B in 55 min, 36% to 95% B in 1 min, 95% B for 5 min, 95% to 4% B in 1 min and 5% B for 8 min, or (2) 4% to 32% B in 190 min, 32% to 95% B in 1 min, 95% B for 2 min, 95% to 4% B in 1 min and 5% B for 6 min. The resulting tandem mass spectra were data-searched using the SequestHT algorithm running on Proteome Discoverer (Thermo Fisher Scientific), in which identified peptides (PSM) are assigned a heavy-to-light ratio value based on integration of precursor ion chromatograms.

### Statistical analyses

Results are presented as mean ± standard deviation (SD). We used one-way analysis of covariance to compare the difference among several groups and significant differences between means were evaluated using Tukey’s test.

## Electronic supplementary material

Supplementary information
