# Supplementary material for: Amnionless-mediated glycosylation is crucial for cell surface targeting of cubilin in renal and intestinal cells
Source: Sci Rep. 2018 Feb 5;8:2351. doi: 10.1038/s41598-018-20731-4 (PMC5799345; doi:10.1038/s41598-018-20731-4)
Supplement: Supplementary file 1 — Supplementary information [file 41598_2018_20731_MOESM1_ESM.pdf]

Supplementary information

Amnionless-mediated glycosylation is crucial for cell surface targeting of cubilin in renal and intestinal cells

Tomohiro Udagawa[1, 2], Yutaka Harita[1], Kenichiro Miura[1], Jun Mitsui[3], Koji L. Ode[4], Shinichi Morishita[5], Seiya Urae[1], Shoichiro Kanda[1], Yuko Kajiho[1], Haruko Tsurumi[1], Hiroki R. Ueda[4], Shoji Tsuji[3], Akihiko Saito[6], Akira Oka[1]

[1] Department of Pediatrics, Graduate School of Medicine, The University of Tokyo, 7-3-1 Hongo, Bunkyo-ku, Tokyo 113-8655, Japan

[2] Department of Pediatrics and Developmental Biology, Tokyo Medical and Dental University, 1-5-45 Yushima, Bunkyo-ku, Tokyo 113-8519, Japan

[3] Department of Neurology, Graduate School of Medicine, The University of Tokyo, 7-3-1 Hongo, Bunkyo-ku, Tokyo 113-8655, Japan

[4] Department of Systems Pharmacology, Graduate School of Medicine, The University of Tokyo, 7-3-1 Hongo, Bunkyo-ku, Tokyo 113-8655, Japan

[5] Department of Computational Biology and Medical Sciences, Graduate School of Frontier Sciences, The University of Tokyo, 5-1-5, Kashiwanoha, Kashiwa-shi, Chiba 277-8561, Japan

[6] Department of Applied Molecular Medicine, Niigata University Graduate School of Medical and Dental Sciences, 1-757, Asahimachidori, Chuo-ku, Niigata-shi, Niigata 951-8510, Japan

Corresponding author:
Yutaka Harita, Department of Pediatrics, Graduate School of Medicine, The University of Tokyo, 7-3-1 Hongo, Bunkyo-ku, Tokyo 113-8655, Japan.
E-mail:haritay-ped@h.u-tokyo.ac.jp.
Tel: +81-3-5800-8659;
Fax: +81-3-3618-4108;

Supplementary information

Case presentation

A 6-year-old Japanese male with megaloblastic anemia and low-molecular weight proteinuria was diagnosed with IGS. Whole exome sequencing and DNA sequences revealed a novel heterozygous mutation in exon 16 of *CUBN* (c.1957G>C (p.Gly653Arg)) (Supplementary Figure 5a). The mutation was de novo and had not been registered in the database of single-nucleotide polymorphisms (dbSNP; www.ncbi.nlm.nih.gov) of the National Center for Biotechnology Information, in the Exome Aggregation Consortium (ExAC)[1], in the Japanese SNP control database established by the National Bioscience Data Base Center (http://gwas.biosciencedbc.jp/snpdb/snp_top.php), nor in the Integrative Japanese Genome Variation Database (https://ijgvd.megabank.tohoku.ac.jp/).

Cubilin Gly653 is located in CUB domain 2 and is well conserved among species (Supplementary Figure S5b). The functionality of *CUBN* G653R was analyzed using the Sorting Intolerant From Tolerant (SIFT) web-based tool (http://sift.jcvi.org), the Polymorphism Phenotyping 2 tool (http://genetics.bwh.harvard.edu/pph2), and Mutation Taster (http://www.mutationtaster.org). All three models predicted *CUBN* G653R as ‘damaging’.

Whole exome analysis

Informed consent for DNA analysis and was obtained from the parents of the patient. The study was performed with the approval from the Ethics Committee of the University of Tokyo. Our study was carried out in accordance with the relevant guidelines and regulations. Genomic DNA was extracted from peripheral white blood cells of the patient and parents using a QIAamp DNA Blood Midi Kit (Qiagen, Hilden, Germany). Exome sequences were enriched using a SureSelect v5+UTRs Kit (Agilent Technologies, CA) from 3 μg of genomic DNA, according to the manufacturer's instructions. The captured DNA samples were subjected to massively parallel sequencing (100-bp paired-end reads) on an Illumina HiSeq2000 sequencing system (Illumina, San Diego, CA). Short reads were aligned to the reference genome (hg19) using BWA [2] with default parameter settings. Single-nucleotide variants and short insertions/deletions were called using SAMtools [3] with default parameter settings. Sanger sequencing was performed to detect *CUBN* or *AMN* and validate the presence of each variant detected by exome sequencing in the patient and his parents.

Semi-quantitative reverse transcriptase and polymerase chain reaction (RT-PCR)

Total RNA was extracted from cultured cells by the Isogen (Nippon Gene, Toyama, Japan). One microgram of each RNA sample was reverse transcribed by SuperScript III RT (Invitrogen, Carlsbad, CA). cDNA was subjected to PCR amplification with specific primers (Supplementary Table 3) and PrimeSTAR Max (Takara, Shiga, Japan). PCR was conducted under the following conditions: 2 min

of initial denaturation at 95°C, 35 cycles (*AMN)*, 33 cycles (*CUBN*), 30 cycles (*LRP2*) and 25 cycles (*GAPDH*) of 98°C for 10 s, an annealing temperature of 55°C for 5 s, 72°C for 10 s and a last extension of 72°C for 2 min. PCR products were separated on an agarose gel and visualised under ultraviolet light by Gelred staining (Nacarai Tesque, Kyoto, Japan).

Proximity Ligation Assay

Proximity ligation assay (PLA) was performed using the Duolink *in situ* PLA kit (Olink Bioscience, Uppsala, Sweden). HEK293T cells were cultured on coverslips coated with collagen type 1, then fixed and permeabilised. After washing in phosphate-buffered saline (PBS), cells were incubated in blocking solution at 37°C for 15 min. After washing, samples were incubated with diluted primary antibodies (rabbit anti-amnionless antibody (1:250, HPA000817) and mouse anti-Flag antibody (1:1000, M2; Sigma-Aldrich, St. Louis, MO, USA) followed by incubation with corresponding secondary antibodies conjugated to PLA oligonucleotide probes (Duolink PLA Rabbit PLUS and PLA Mouse MINUS proximity probes, Uppsala, Sweden) for 1 h at 37°C. Hybridisation and ligation of connector oligonucleotides, rolling-circle amplification and detection of amplified DNA products using detection reagent Red were performed using the Duolink detection reagent kit according to the manufacturer's protocol. The samples were co-stained with DAPI before mounting.

Internalisation assay

Endocytosis of mini-cubilin was analysed by studying the internalisation of anti-Flag antibody in transfected MDCK cells expressing WT or G653R mini-cubilin–Flag, with or without WT Amnionless–mycGFP. Cells were incubated with the anti-Flag antibody, diluted 1:1000 in culture medium, for 1 h on ice and were subsequently transferred to either 4°C or 37°C and continuously incubated for 30 min to allow internalisation of bound antibody. After the incubation period, cells were washed in PBS, fixed in 2% paraformaldehyde in PBS for 5 min and permeabilised in 0.1% Triton X-100 in PBS for 10 min. Cells were stained with Alexa Fluor 555 goat anti-mouse IgG1 in PBS with 1% fetal bovine serum for 1 h at room temperature. Slides were mounted in a medium containing DAPI.

Supplementary Reference

1. Lek M, Karczewski KJ, Minikel EV, *et al*. Analysis of protein-coding genetic variation in 60,706 humans. *Nature* 2016; 536: 285-291.

2. Li H, Durbin R. Fast and accurate short read alignment with Burrows-Wheeler transform. *Bioinformatics* 2009; 25: 1754-1760.

3. Li H, Handsaker B, Wysoker A, *et al*. The Sequence Alignment/Map format and SAMtools. *Bioinformatics* 2009; 25: 2078-2079.

Supplementary Figure S1

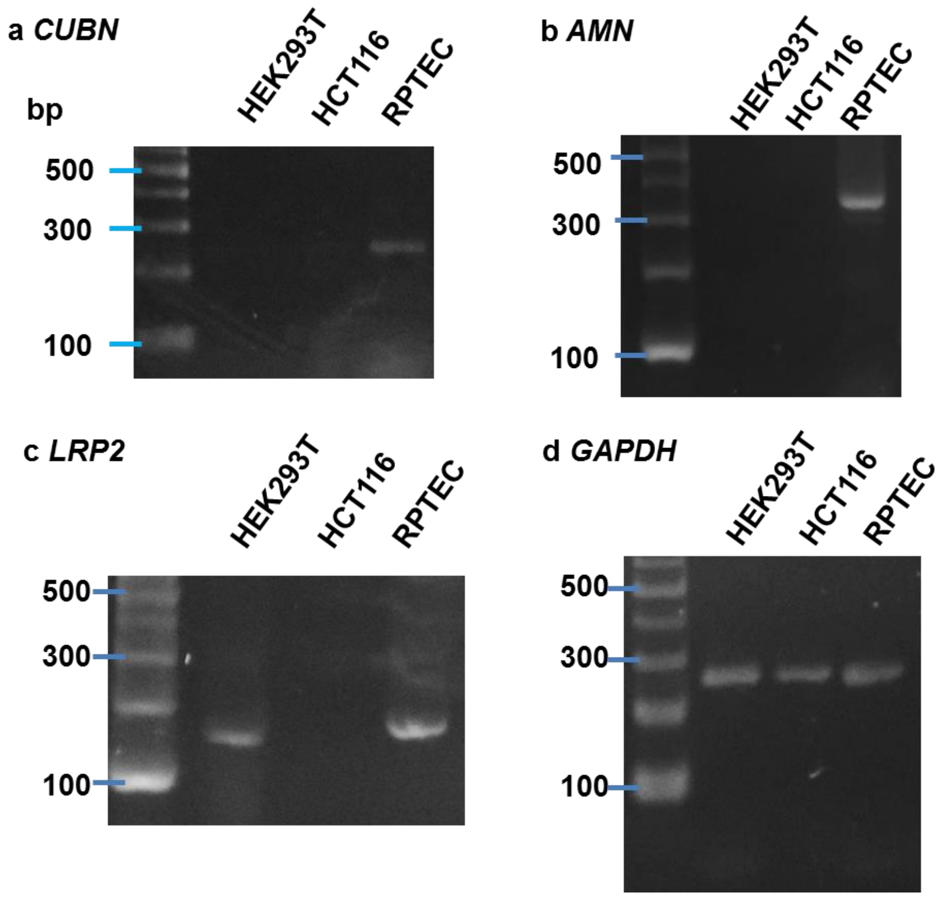

Supplementary Figure S1. Endogenous transcript of *CUBN*, *AMN*, *LRP2*, and *GAPDH* in human cultured cells analyzed by RT-PCR

Total RNA was extracted from HEK293T cells, HCT116 cells, and human renal proximal tubule epithelial cells (RPTECs). Semi quantitative RT-PCR for *CUBN* **(a)**, *AMN* **(b)**, *LRP2* (megalin) **(c)**, and *GAPDH* **(d)** was carried out using 50ng of RNA extracted from cells. The primers used for PCR amplification and PCR conditions were described in Supplementary Table 3.

Supplementary Figure S2

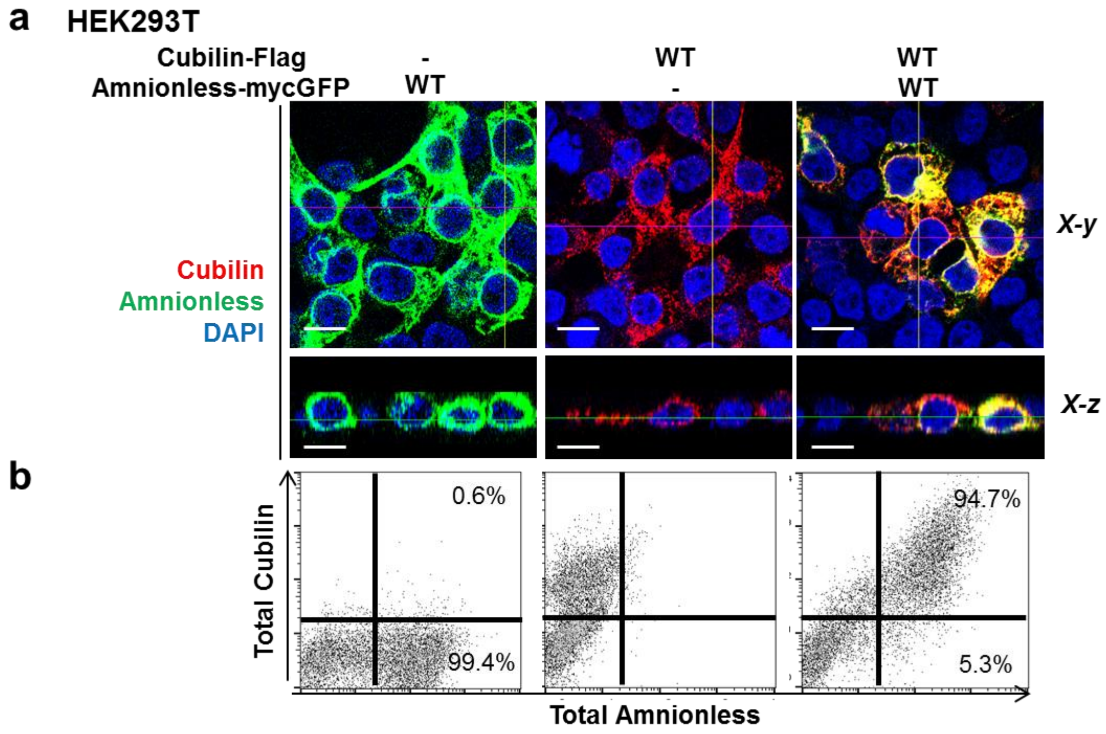

Supplementary Figure S2. Expression of exogenous cubilin and amnionless in transfected HEK293T cells

(a) HEK293T cells transfected with the indicated vectors were fixed, permeabilised, and stained with mouse monoclonal anti-Flag antibody (red). GFP-tagged amnionless is shown in green and DAPI nuclear staining in blue. Representative confocal sections are taken from the middle height of cells (*top*) and *X-Z* vertical sections (*bottom*) (Scale bar: 10 µm) **(b)** Expression of cubilin and amnionless in permeabilised HEK293T cells transfected with the indicated vectors were analysed by flow cytometry.

Supplementary Figure S3

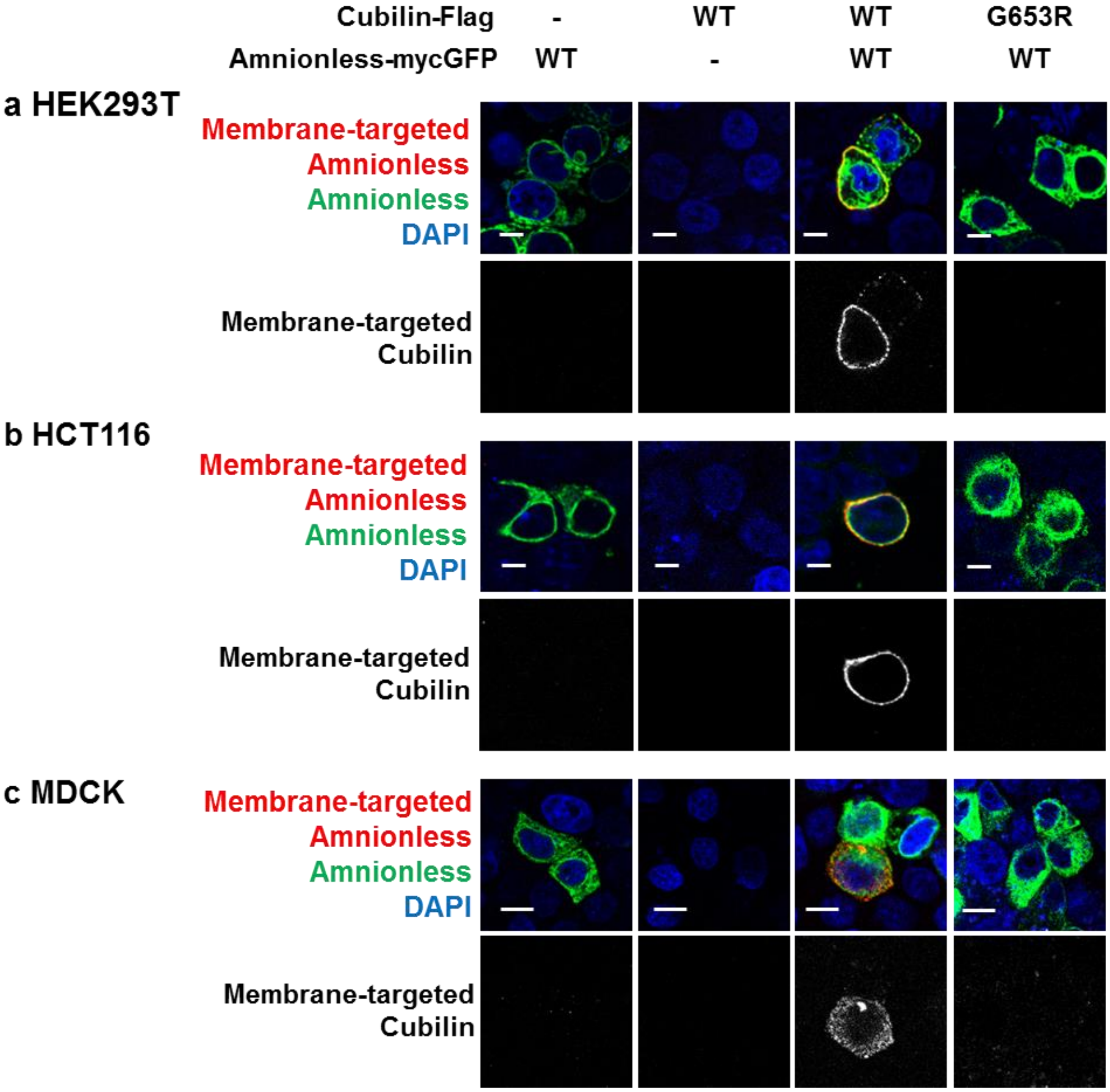

Supplementary Figure S3. Cubilin-dependent plasma membrane targeting of amnionless in transfected cultured cells

(a-c) HEK293T **(a)**, HCT116 **(b)** and MDCK cells **(c)** transfected with wild-type cubilin-flag and wild-amnionless-mycGFP were fixed and stained for membrane-targeted amnionless (red). GFP-tagged amnionless is shown in green and DAPI nuclear staining in blue. Representative confocal sections are taken from the middle height of cells. (Scale bar: 10 μm)

Supplementary Figure S4

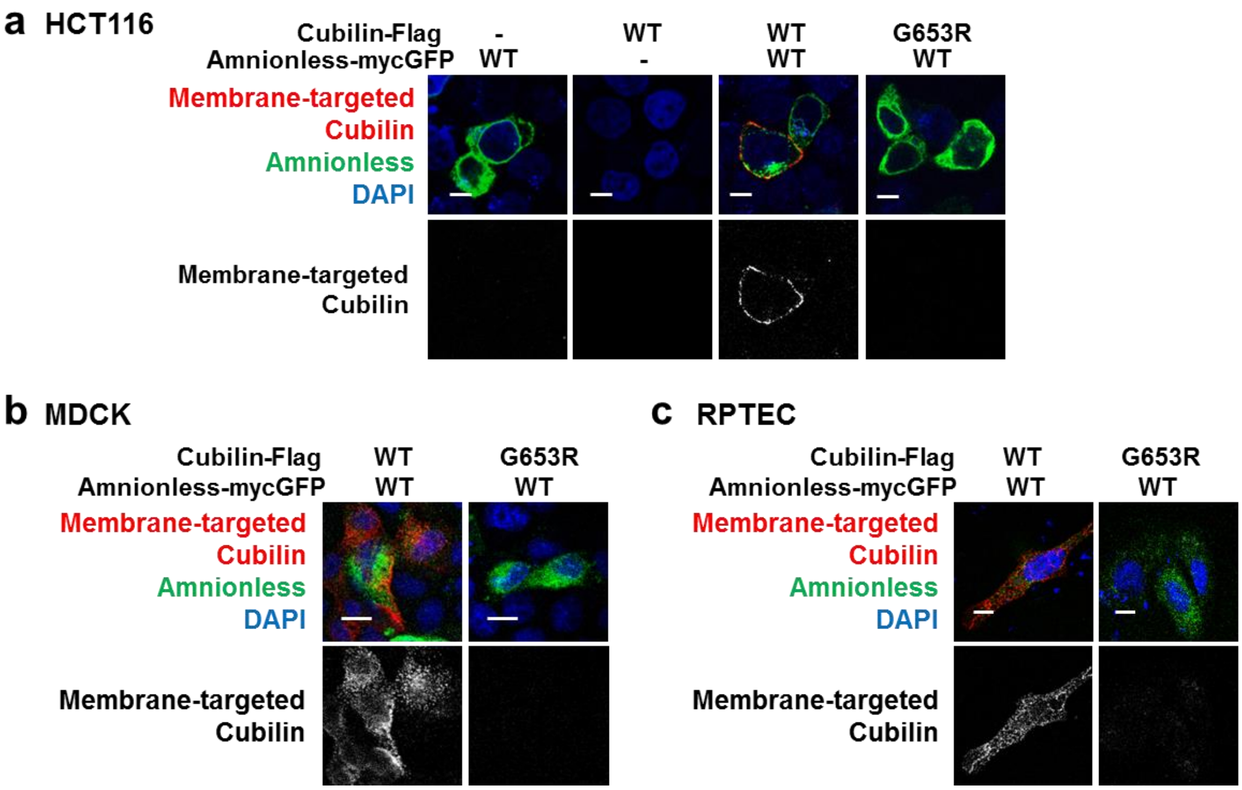

Supplementary Figure S4. G653R cubilin inhibited amnionless-dependent plasma membrane targeting of cubilin in transfected cultured cells

HCT116 cells **(a)**, MDCK cells **(b)**, and RPTECs **(c)** transfected with indicated vectors were fixed and stained for membrane-targeted cubilin (red). GFP-tagged amnionless is shown in green and DAPI nuclear staining in blue. Representative confocal sections are taken from the middle height of cells. (Scale bar: 10 μm)

Supplementary Figure S5

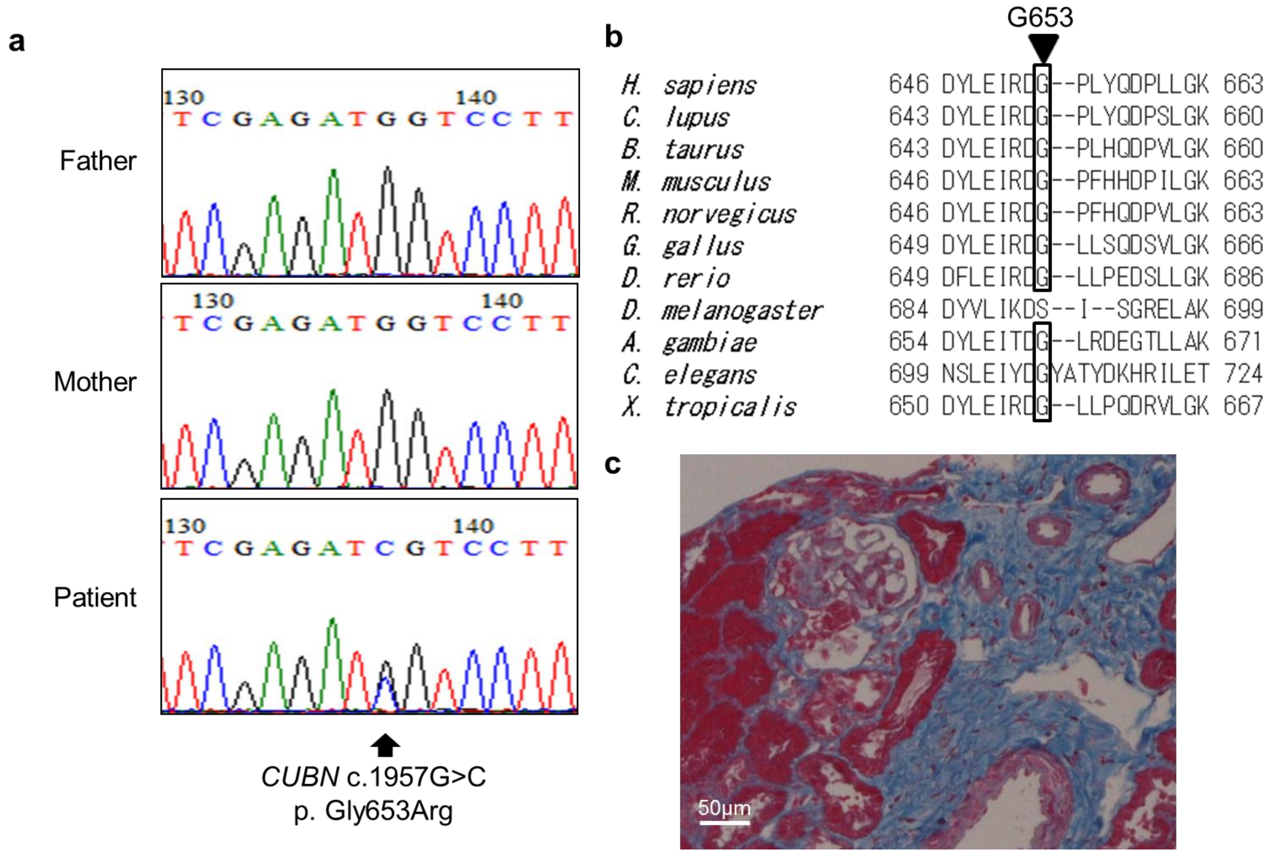

Supplementary Figure S5. ***CUBN*** **G653R mutation in a boy with IGS**

(a) Chromatograph of *CUBN* in exon 16 in the patient with IGS and his parents. **(b)** G653 is highly conserved across species. **(c)** Renal histology of the patient with IGS. (Masson's trichrome stain)

Supplementary Figure S6

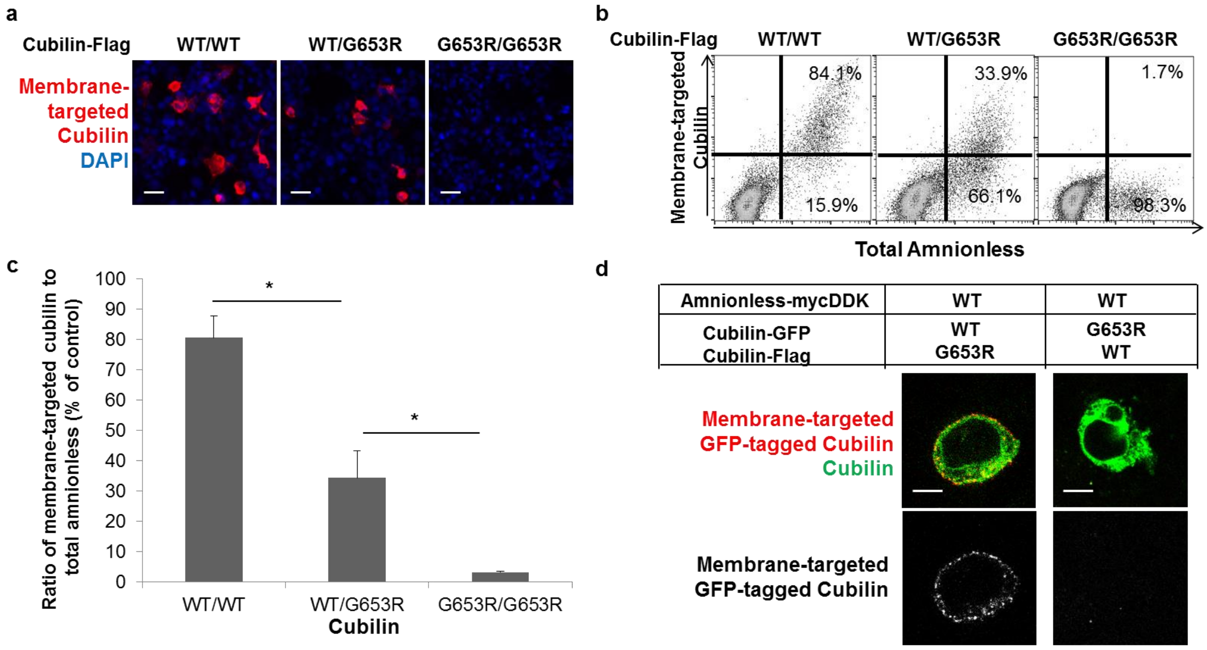

Supplementary Figure S6. *CUBN* G653R mutation abrogated amnionless-dependent membrane expression of cubilin in a dose-dependent manner

(a) Non-permeabilised HEK293T cells cotransfected with wild-type (WT) amnionless-mycGFP and WT cubilin, mixture of WT and G653R cubilin, or G653R cubilin vectors were stained for membrane-targeted cubilin. (Scale bar: 20 μm) **(b)** Membrane expression of cubilin in **(a)** was analysed by flow cytometry. **(c)** The ratio of cells with amnionless-dependent membrane-targeted cubilin to amnionless-expressing cells was analysed by flow cytometry. Statistical analysis was carried out using the Student's *t*-test. * $P < 0.01$. N = 3. **(d)** Non-permeabilised HEK293T cells cotransfected with both amnionless-mycDDK and mixture of WT cubilin-GFP and G653R cubilin-flag (left panel), or both amnionless-mycDDK and mixture of WT cubilin-flag and G653R cubilin-GFP (right panel) vectors were fixed and stained for membrane-targeted cubilin by monoclonal anti-GFP antibody in red. (Scale bar; 10 μm)

Supplementary Figure S7

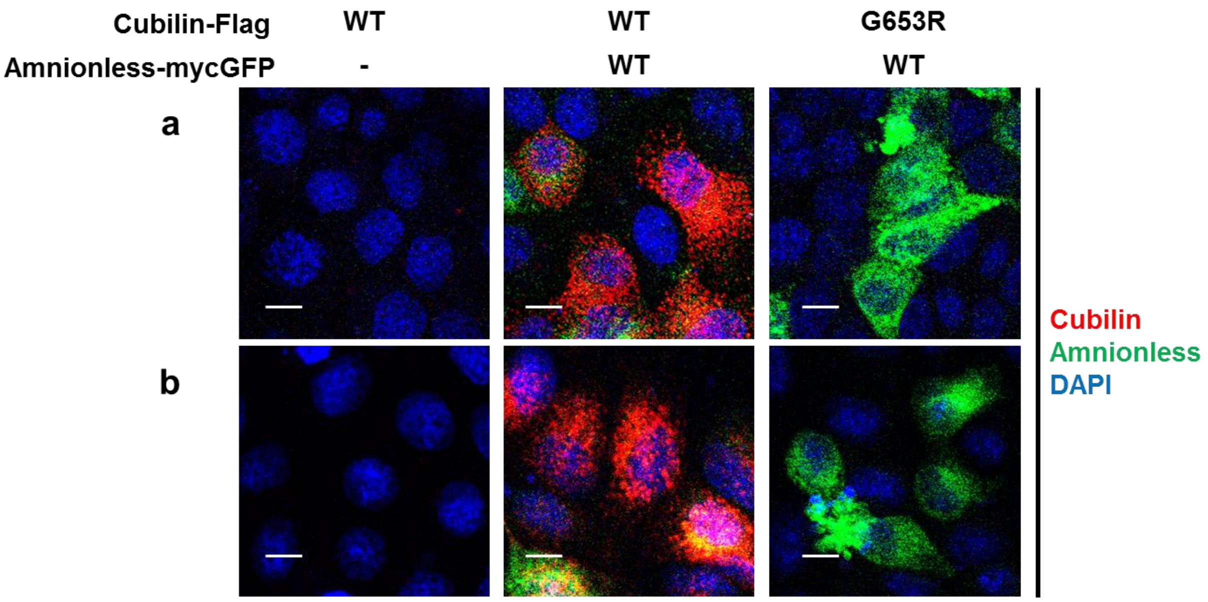

Supplementary Figure S7. Internalisation of cubilin is abrogated by *CUBN* G653R mutation

MDCK cells transiently transfected with the indicated cubilin and amnionless vectors were labeled with anti-Flag antibody at 4°C, and incubated at either 4°C **(a)** or 37°C **(b)**. Following fixation, cells were permeabilised and stained with Alexa555 conjugated secondary antibody (red). GFP-tagged amnionless is shown in green and DAPI nuclear staining in blue. Representative confocal sections are taken from the middle height of cells. (Scale bar: 10 µm)

Supplementary Figure S8

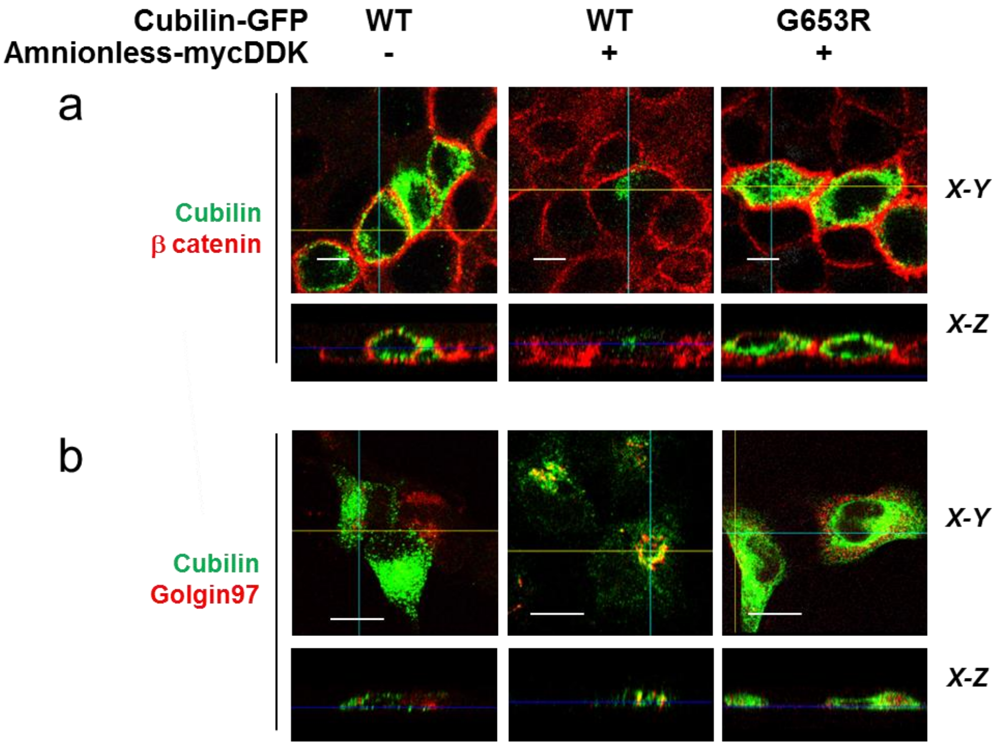

Supplementary Figure S8. Amnionless-dependent trafficking of cubilin to Golgi apparatus
MDCK cells transfected with indicated vector were fixed, permeabilised and labeled for β-catenin **(a)** and golgin 97 **(b)** followed by Alexa Fluor 555-conjugated secondary antibody (red). GFP-tagged cubilin is shown in green and DAPI nuclear staining in blue. Pictures are confocal sections taken from the middle height of cells. (Scale bar: 10 μm)

Supplementary Figure S9

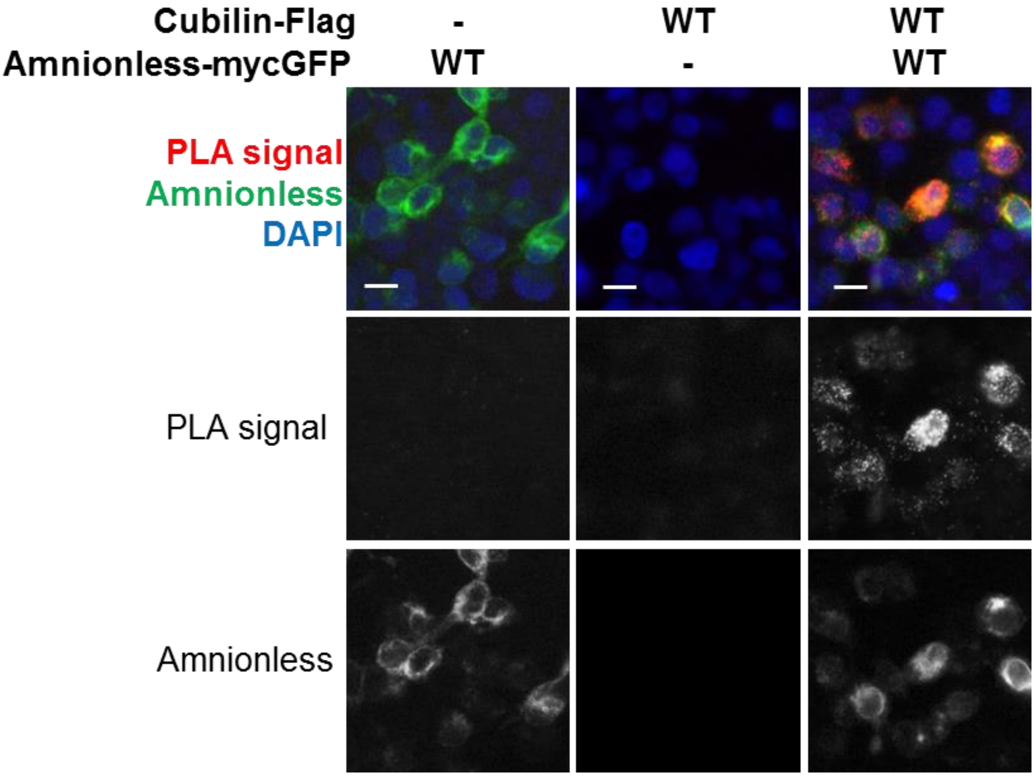

Supplementary Figure S9. Detection of interaction of cubilin and amnionless by in situ proximity ligation assay in HEK293T cells.

Rabbit anti-amnionless and mouse anti-flag antibodies were combined with secondary PLA probes (Olink Bioscience). GFP-tagged amnionless is shown in green and DAPI nuclear staining in blue. The interaction events are visible as red dots. (Scale bar, 10 μm)

Supplementary Figure S10

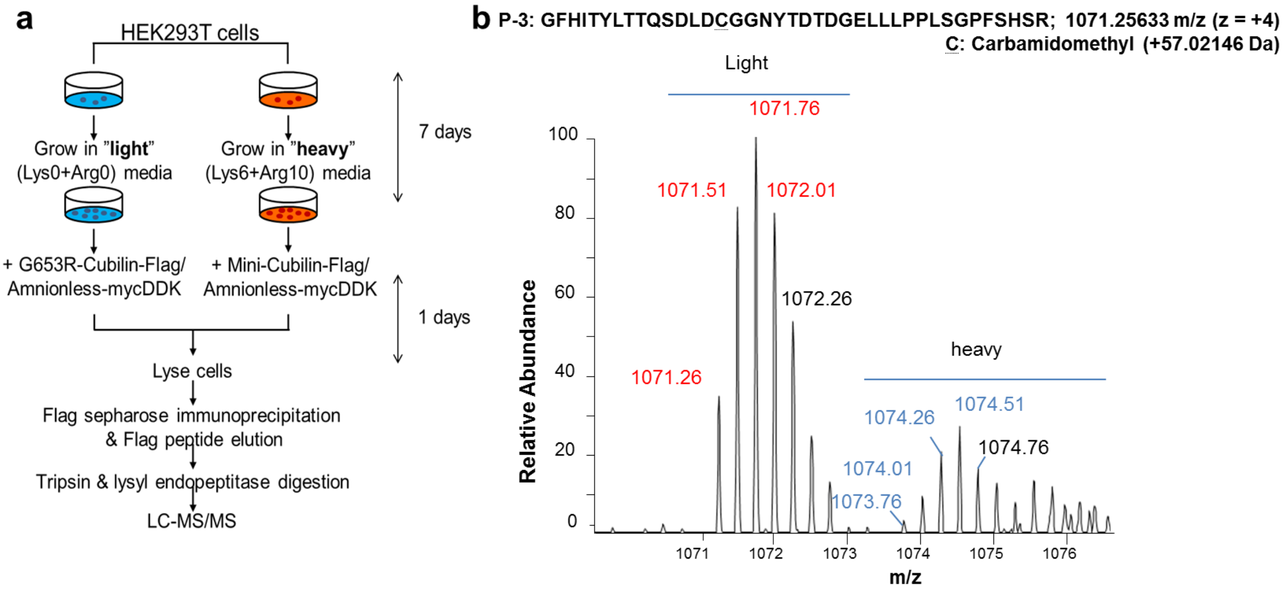

Supplementary Figure S10. G653R mutation altered modification of a cubilin peptide (P-3)
(a) A schematic diagram of SILAC labeling and proteome analysis. HEK293T cells transfected with G653R cubilin-Flag and wildtype amnionless or wild-typed cubilin-Flag and amnionless-mycDDK were labeled with light (L-$^{12}C_6{}^{14}N_4$-Arg and L-$^{12}C_6$-Lys) or heavy (L-$^{13}C_6{}^{15}N_4$-Arg and L-$^{13}C_6$-Lys) isotope-labeled amino acids respectively. Equal amounts of cell lysate from ‘light’ and ‘heavy’ cells were mixed and immunoprecipitated with anti-Flag antibody. After trypsin/lysyl endopeptidase digestion, peptides were analyzed by LC-MS/MS. **(b)** Representative spectral data of cubilin peptides including N-glycosylation site (P-3) is shown.

Supplementary Figure S11

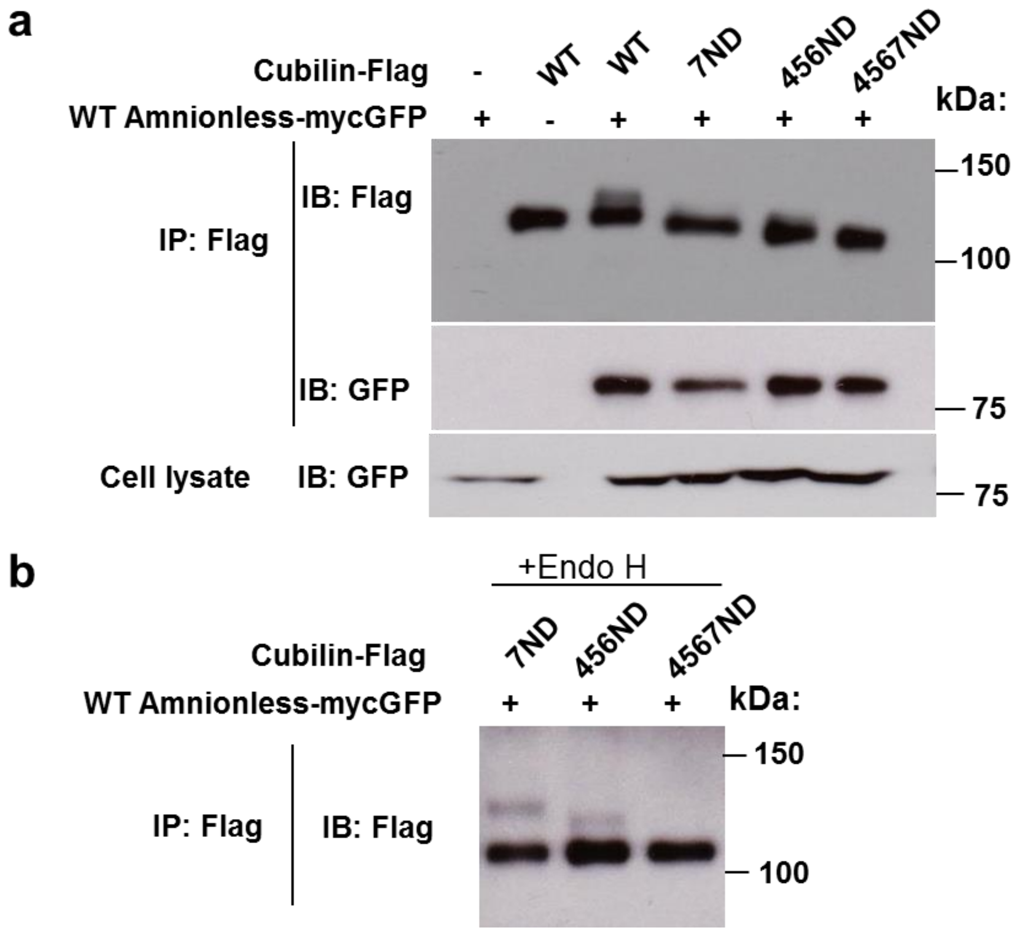

Supplementary Figure S11 The combined of N-glycosylation at several Asn residues is essential for mature glycosylation of cubilin.

(a) HEK293T cells were transfected with the indicated vectors, and anti-Flag immunoprecipitates and cell lysates were analyzed by western blotting with monoclonal anti-flag antibody for cubilin and monoclonal anti-GFP antibody for amnionless. **(b)** HEK293T cells were transiently transfected with the indicated vectors, and anti-flag immunoprecipitates were treated with endoglycosidase H (endo H), and analyzed by western blotting.

Supplementary Figure 12

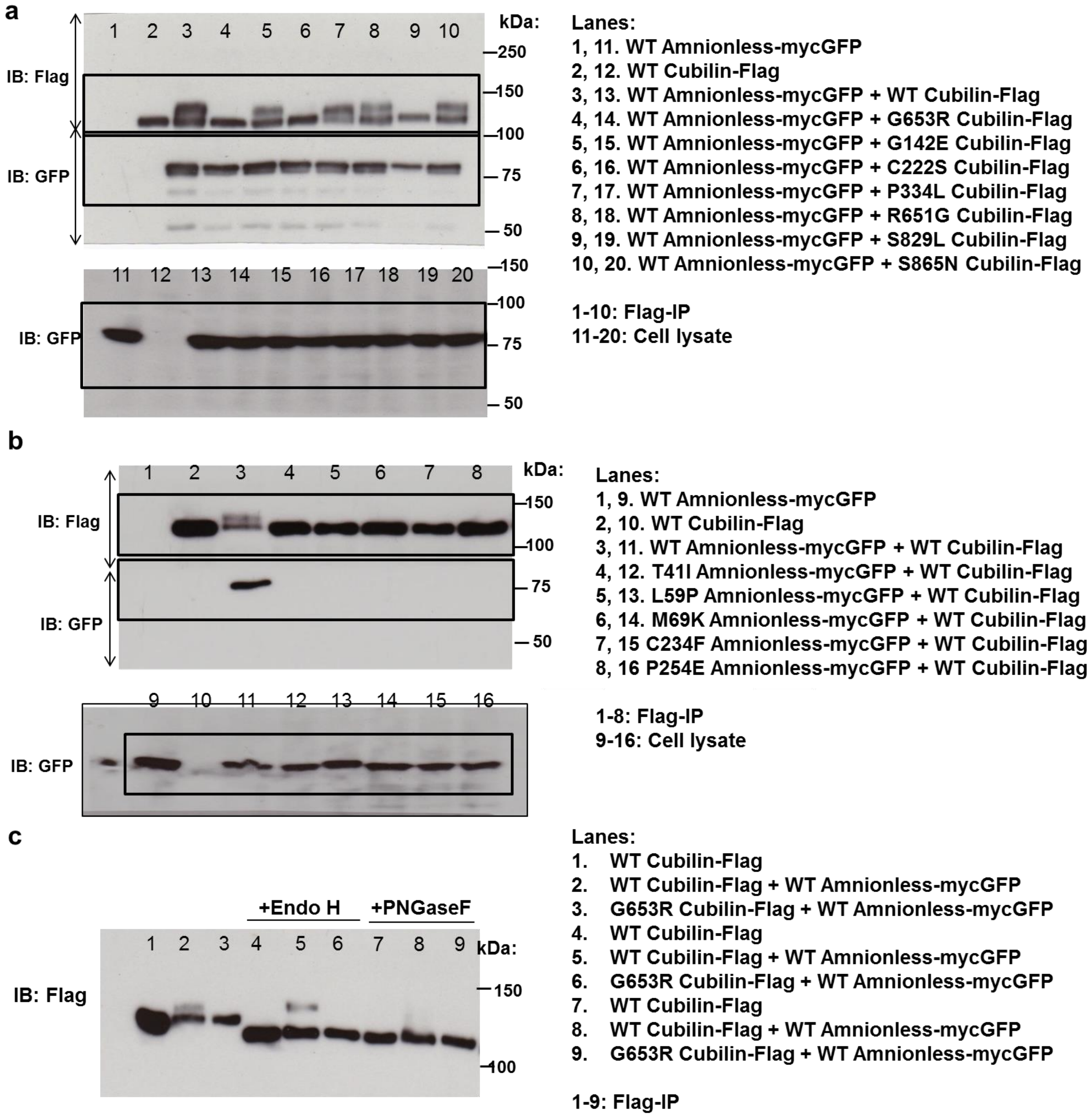

Supplementary Figure 12. Full length immunoblots shown in Figure 5

Boxes indicated lanes which was used in Figure 5.

Supplementary Figure 13

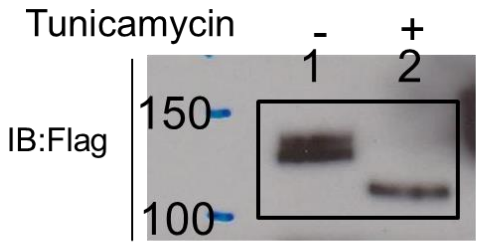

Lanes:

1. **WT Cubilin-Flag+ WT Amnionless-mycGFP**
2. **WT Cubilin-Flag + WT Amnionless-mycGFP**

1-2: Flag-IP

Supplementary Figure 13. Full length immunoblots shown in Figure 6e

Box indicated lanes which was used in Figure 6e

Supplementary Table 1

CUBN mutations

Description		Protein region	Genotype	Allele frequency		Reference
human	rat			ExAC	gnomAD	
p.Gly145Glu	p.Gly142Glu	EGF repeats	Homo	ND	ND	16
p.Cys225Ser	p.Cys222Ser	EGF repeats	Homo	ND	ND	16
p.Pro337Leu	p.Pro334Leu	CUB domain 1	Comp het	0.000126 (European (Non-Finnish))	0.0002210 (European (Non-Finnish))	16, 17
p.Arg651Gly	p.Arg651Gly	CUB domain 2	Comp het ?	0.0158 (east asian)	0.01658 (east asian)	16
p.Ser829Leu	p.Ser829Leu	CUB domain 4	Comp het ?	ND	0.000008977 (European (Non-Finnish))	16
p.Ser865Asn	p.Ser865Asn	CUB domain 4	Homo or comp het	0.01065 (European (Non-Finnish))	0.01169 (European (Non-Finnish))	16
p.Gly653Ala	p.Gly653Ala	CUB domain 2		3.028e-05 (European (Non-Finnish))	0.00001794 (European (Non-Finnish))	
p.Gly653Ser	p.Gly653Ser	CUB domain 2		0 (None)	0.0008146 (European (Finnish))	

Supplementary Table 1 List of missense mutations of *CUBN* in Imerslund–Gräsbeck syndrome patients

List of human *CUBN* mutations previously reported and corresponding amino acids alterations in Rat homolog are shown. comp het: compound heterozygous mutation.

Supplementary Table 2

AMN mutations					
Description	Protein region	Genotype	Allele frequency		Reference
			ExAC	gnomAD	
p.Thr41Ile	Extracellular domain	Homo	5e-06 (European (Non-Finnish))	0.00005578 (European (Non-Finnish))	16
p.Leu59Pro	Extracellular domain	Homo	ND	ND	16
p.Met69Lys	Extracellular domain	Comp het	ND	0.00001582 (European (Non-Finnish))	18
p.Cys234Phe	Extracellular domain	Comp het	ND	ND	16
p.Pro254Glu	Extracellular domain	Homo	ND	ND	16

Supplementary Table 2 List of missense mutations of *AMN* in Imerslund–Gräsbeck syndrome patients

List of human *AMN* mutations previously reported are shown. comp het: compound heterozygous mutation.

Supplementary Table 3

Gene	Primer	Sequence(5’-3’)	Product length
CUBN	F	GTGGAACCTGCCTCAATCTGC	248 bp
	R	GACACAGCGTGCCACAGAAC	
AMN	F	AACTGGAGCCAGAACCGGAC	346 bp
	R	AGGAGGCACTAGGCGGAAAG	
LRP2	F	AATTGAGCACAGCACCTTTGA	151bp
	R	GCTTTCCTGACTCGAATAATG	
GAPDH	F	ATCATCCCTGCCTCTACTGG	257 bp
	R	TGGGTGTCGCTGTTGAAGTC	

Supplementary Table 3 Oligo-primer pairs used for RT-PCR amplification
